# Impact of Hfq on Global Gene Expression and Virulence in *Klebsiella pneumoniae*

**DOI:** 10.1371/journal.pone.0022248

**Published:** 2011-07-14

**Authors:** Ming-Ko Chiang, Min-Chi Lu, Li-Cheng Liu, Ching-Ting Lin, Yi-Chyi Lai

**Affiliations:** 1 Department of Life Science, National Chung-Cheng University, Chia-Yi, Taiwan; 2 Division of Infectious Diseases, Department of Internal Medicine, Chung-Shan Medical University Hospital, Taichung, Taiwan; 3 Department of Microbiology and Immunology, Chung-Shan Medical University, Taichung, Taiwan; 4 Institute of Medicine, Chung-Shan Medical University, Taichung, Taiwan; 5 School of Chinese Medicine, China Medical University, Taichung, Taiwan; Universite de la Mediterranee, France

## Abstract

*Klebsiella pneumoniae* is responsible for a wide range of clinical symptoms. How this bacterium adapts itself to ever-changing host milieu is still a mystery. Recently, small non-coding RNAs (sRNAs) have received considerable attention for their functions in fine-tuning gene expression at a post-transcriptional level to promote bacterial adaptation. Here we demonstrate that Hfq, an RNA-binding protein, which facilitates interactions between sRNAs and their mRNA targets, is critical for *K. pneumoniae* virulence. A *K. pneumoniae* mutant lacking *hfq* (Δ*hfq*) failed to disseminate into extra-intestinal organs and was attenuated on induction of a systemic infection in a mouse model. The absence of Hfq was associated with alteration in composition of envelope proteins, increased production of capsular polysaccharides, and decreased resistance to H_2_O_2_, heat shock, and UV irradiation. Microarray-based transcriptome analyses revealed that 897 genes involved in numerous cellular processes were deregulated in the Δ*hfq* strain. Interestingly, Hfq appeared to govern expression of many genes indirectly by affecting sigma factor RpoS and RpoE, since 19.5% (175/897) and 17.3% (155/897) of Hfq-dependent genes belong to the RpoE- and RpoS-regulon, respectively. These results indicate that Hfq regulates global gene expression at multiple levels to modulate the physiological fitness and virulence potential of *K. pneumoniae*.

## Introduction

Since *Klebsiella* was first identified as a pathogen of pneumonia in 1882, the remarkable ability of *K. pneumoniae* to cause a wild range of human diseases, from urinary tract infections to life-threatening systemic infections [1], has attracted increasing attention to the pathogenesis of this bacterium. Not solely confined inside the human host, *K. pneumoniae* has a great capacity for adaptation to diverse environments, including the surface water, sewage, soil, intestinal tracts of mammals [1], and even the interior of plants [2]. How *K. pneumoniae* responds to environmental changes and thus adapts itself to a specific niche becomes an interesting question. Nevertheless, our knowledge with regard to the regulatory mechanisms which this bacterium utilizes to ensure its survival upon different conditions is very limited.

An ever-increasing number and variety of small non-coding RNAs (sRNAs) are being identified to serve regulatory functions in bacteria. Numerous cellular processes, such as iron homeostasis [3], outer membrane proteins (OMPs) biogenesis [4], sugar metabolism [5], quorum sensing [6] and various stress responses [7], are subject to the post-transcriptional control exerted by sRNAs. At present, most characterized sRNAs regulate gene expression by basepairing with mRNAs. While some sRNAs are cis-encoded having the potential to basepair mRNAs with long stretches, the majority of regulatory sRNAs in Gram-negative bacteria are trans-encoded and share limited complementarity with their target mRNAs [8]. The trans-acting sRNAs are functionally analogous to eukaryotic miRNAs that usually exert negative regulation by repress protein levels through translation inhibition, mRNA degradation, or both [9]. In many cases, because of the limited complementarity, the trans-acting sRNAs mediated regulation requires the chaperone protein Hfq to facilitate RNA-RNA interactions.

Hfq assembles into homohexameric rings which are structurally similar to those formed by Sm and Sm-like proteins in eukaryotic cells [10]. Besides enhancing the formation of sRNA-mRNA duplex, Hfq contributes to RNA regulation through interacting with RNA turnover enzymes, including RNase E, polynucleotide phosphorylase, and poly (A) polymerase [11]. Hfq has a broad and diverse impact on bacterial physiology and virulence beyond its original role as a host factor required for replication of Qβ RNA bacteriophage [12]. Defects including reduced growth, impaired resistance to various stresses, and altered virulence are detected in *E. coli* lacking *hfq* [13]. It has also been shown that virulence of several pathogenic bacteria, including *Brucella abortus* [14], *Francisella tularensis* [15], *Vibrio cholera* [16], *Listeria monocytogenes* [17], *Legionella pneumophila* [18], *Pseudomonas aeruginosa* [19], *Yersinia* [20], [21], *Salmonella* Typhimurium [22], and uropathogenic *E. coli* [23], were significantly attenuated by *hfq* mutations.

Recently, by sequence analysis, we have identified an *hfq* homologue and sRNAs from *K. pneumoniae* genomes. The presence of these homologues in various strains of *K. pneumoniae*, including NTUH-K2044 (NC_012731), MGH78578 (NC_009648), and 342 (NC_011283) suggests that this pathogen also utilizes the post-transcriptional regulation mediated by Hfq to control various cellular processes. However, the role of Hfq-sRNA mediated regulation in *K. pneumoniae* is yet to be defined. In this study, we aimed to understand how Hfq contributed to the control of gene expression and the pathogenesis in *K. pneumoniae*. An *hfq* deletion mutant was generated in *K. pneumoniae* CG43S. The loss of *hfq* attenuated *K. pneumoniae* virulence in a mouse model, as well as altered physiological characteristics of *K. pneumoniae*, including production of capsular polysaccharides, stress tolerance, and homeostasis of envelope. The microarray data demonstrated that the expression of almost a fifth of *K. pneumoniae* genes was drastically deregulated. It also suggested that beside directly regulating individual genes expression at the post-transcriptional level, by affecting sigma factor RpoS and RpoE, Hfq positioned itself at the upper level of the gene regulatory hierarchy that control the physiological fitness and virulence potential of *K. pneumoniae*.

## Results

### Deletion of *hfq* attenuated *K. pneumoniae* virulence

**Figure 1:**
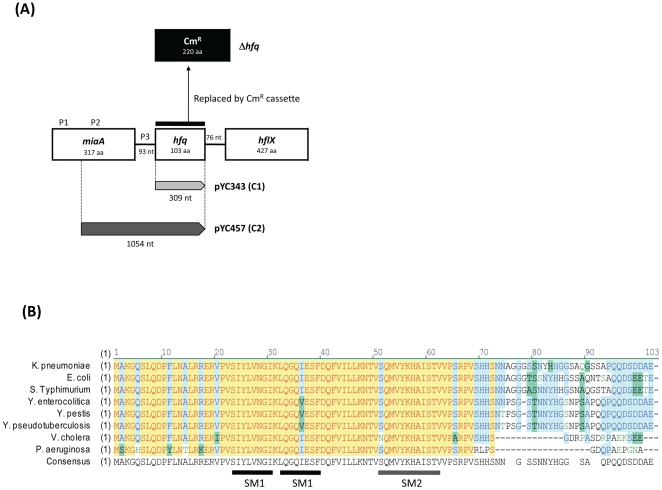
*K. pneumoniae hfq*. (**A**) Genomic organization of *hfq* gene in *K. pneumoniae*. In the Δ*hfq* strain, the coding region of *hfq* was deleted and replaced by a chloramphenicol cassette amplified from pACYC184. The coding region of *hfq*, represented as a light grey bar, was cloned to pBAD202, under the control of arabinose-inducible promoter, to give the complementation plasmid pYC343. The grey bar indicates the region of *hfq* together with its native promoter cloned to pACYC184 yielding the complementation plasmid pYC457. Three promoter regions of *hfq* are indicated as P1, P2, and P3, respectively. (**B**) Alignment of *hfq* sequences of the Gram-negative pathogens with their Hfq functionally identified. The highly conserved SM1 and SM2 motifs are indicated as black and grey lines, respectively.

The *hfq* gene is located in clockwise orientation at bps 446148–446456 in the genome of *K. pneumoniae* strain NTHU-K2044 [24]. As in *E. coli*, it is located in the *miaA-hfq-hflX* cluster with three promoter regions as indicated in Fig. 1A. The nucleotide sequence of *hfq* region in *K. pneumoniae* CG43S is identical to that in strain NTHU-K2044. Additionally, *K. pneumoniae* Hfq protein shares 85.4% sequence identity with its homologue in *E. coli*, and just like *Enterobacteriaceae* Hfq protein, it has the conserved Sm1 and Sm2 motifs (Fig. 1B). To explore the physiological role of Hfq, an *hfq* deletion mutant, named Δ*hfq*, was constructed in the genetic background of *K. pneumoniae* CG43S. Furthermore, two trans-complemented strains, Δ*hfq*-C1 and Δ*hfq*-C2, which carried the *hfq* gene under the control of pBAD promoter and its native promoters, respectively, were generated as described in Materials and Methods (Fig. 1A).

**Figure 2:**
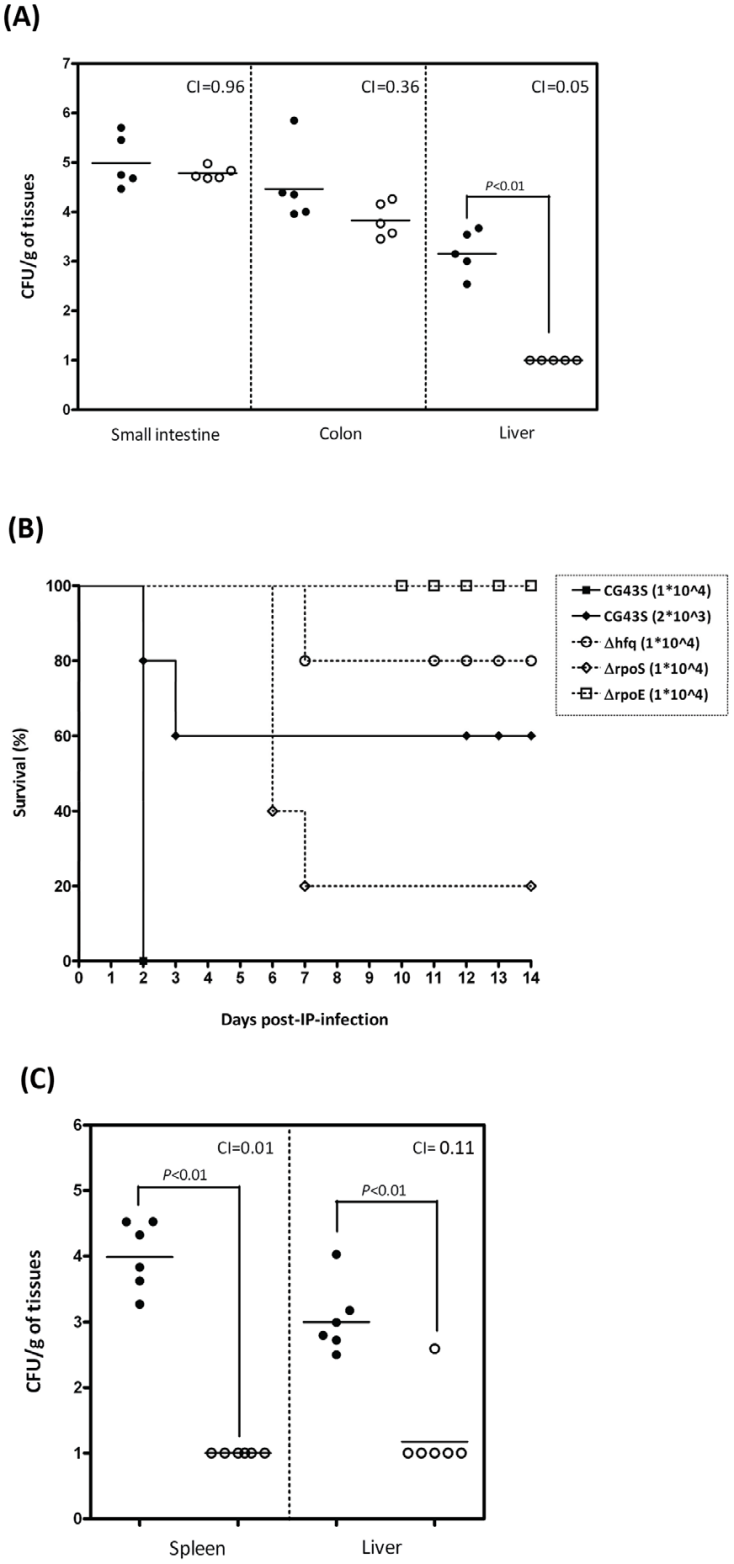
*K. pneumoniae* virulence attenuated by the absence of *hfq*. (**A**) Bacterial loads in small intestine, colon, and liver determined at 48 h after oral inoculation with suspension containing equal amount of *K. pneumoniae* CG43S (5×10^8^ CFU) and Δ*hfq* (5×10^8^ CFU). Filled and open circles represent CG43S and Δ*hfq* retrieved from five BALB/c mice, respectively. (**B**) Survival of *K. pneumoniae*-infected mice. Groups of five mice were inoculated by intraperitoneal injection with 2×10^3^ CFU of CG43S (filled diamonds), or with 1×10^4^ CFU of CG43S (filled squares), Δ*hfq* (open circles), Δ*rpoS* (open diamonds), or Δ*rpoE* (open squares), and monitored for 14 days. (**C**) Groups of six mice were inoculated intraperitoneally with bacterial suspension containing equal amount of *K. pneumoniae* CG43S (1×10^3^ CFU) and Δ*hfq* (1×10^3^ CFU). Bacterial loads of CG43S (filled circles) and Δ*hfq* (open circles) in spleen and liver were determined at 6 h post-inoculation. Horizontal bars indicate geometric means. The limit of detection was approximately 10 CFU. Samples which yielded no colonies were plotted having the value as 10 CFU g^−1^ tissues. Competitive index is defined as Δ*hfq*_ output_/CG43S_output_ ÷ Δ*hfq*_ input_/CG43S_input_. The indicated *P* values were determined using the Student’s *t*-test.

To examine whether Hfq was critical for virulence of *K. pneumoniae*, competitive assays were performed in 8-week-old male BALB/c mice by orally inoculating them with the bacterial mixture containing equal amount of Δ*hfq* and the parental strain CG43S [25]. At 48 hour post-inoculation (hpi), colonization of the small intestine by Δ*hfq* was comparable to that by CG43S. However, in the colon Δ*hfq* was out-competed by CG43S with a CI value of 0.36 (Fig. 2A). While all the CG43S-infected mice had a bacterial burden approaching 10^2^ to 10^4^ CFU in the liver, Δ*hfq* was undetectable at the same time point (Fig. 2A). Diminishment of Δ*hfq* in the liver suggested that the disseminating ability of *K. pneumoniae* to extra-intestinal organs was abolished by the absence of *hfq*. To further investigate whether Hfq was involved in a systemic infection of *K. pneumoniae*, groups of mice were intraperitoneally inoculated with Δ*hfq* or CG43S and their survival was monitored for two weeks. The intraperitoneal inoculation method allowed the infection to bypass the colonization step of *K. pneumoniae* in the small intestine. When mice were inoculated with 10^4^ CFU of CG43S, all of them died by day 2 (filled squares, Fig. 2B). Even when the inoculums was decreased to 10^3^ CFU, 60% of the mice still succumbed to the infection of CG43S (filled diamonds, Fig. 2B). On the other hand, when mice were inoculated with 10^4^ CFU of Δ*hfq*, 80% of the mice survived the experimental period (open circles, Fig. 2B). An involvement of *hfq* in a systemic *K. pneumoniae* infection was further supported by *in vivo* competition results. As shown in Fig. 2C, Δ*hfq* was out-competed by CG43S at 6 h after intraperitoneal inoculation with the average CI values of 0.11 and 0.01 in the liver and spleen, respectively (Fig. 2C). The significant decline in the *in vivo* CI values might not be due to an inability to compete under nutrient-limiting conditions, as the growth of Δ*hfq* approximated to that of CG43S with the *in vitro* CI value of 0.82 in M9 medium.

### Hfq affected global gene expression

**Figure 3:**
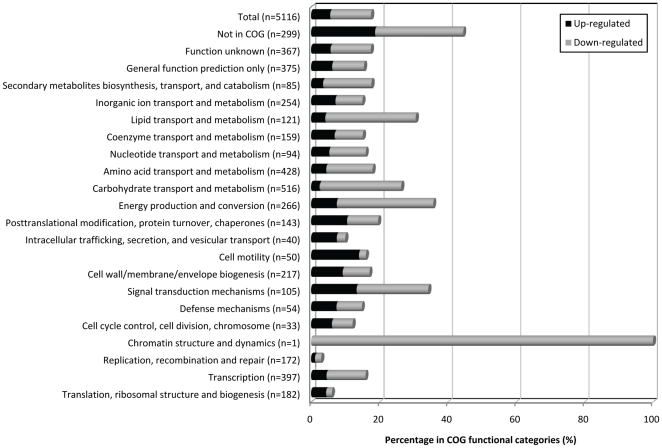
Functional classification of Hfq-dependent genes. Microarray-based transcriptome analyses revealed 897 ORFs that are significantly deregulated in Δ*hfq* as compared to that in CG43S. Based on the genome project of *K. pneumoniae* NTHU-K2044 (NC012731;[24]), the 897 Hfq-dependent genes were grouped into different functional classes. The values represent the percentage of genes affected by Hfq in Δ*hfq* versus CG43S within the respective class. Black bars: up-regulated genes; grey bars: down-regulated genes.

Previous reports have shown that Hfq contributes to the regulation of numerous cellular pathways in *E. coli* [23], [26], [27]. To gain insight into the genes whose expression was regulated by Hfq in *K. pneumoniae*, DNA microarray experiments were performed to compare the transcriptome of Δ*hfq* with that of CG43S. Probes were made from the RNA of *K. pneumoniae* which were grown to log-phase in LB medium at 37^o^C. Of 5,024 genes in the *K. pneumoniae* genome, 897 genes (approximately 18%) showed a >1.5log_2_ fold change (2.83-fold) in transcript abundance in Δ*hfq* when compared to that in CG43S. Among the 897 Hfq-dependent genes, down-regulated genes (n = 610) were more than twice as up-regulated genes (n = 287), suggesting that Hfq-mediated regulation in *K. pneumoniae* was more frequently positive than negative. Based on the genome annotation of NTHU-K2044 strain (NC012731;[24]), the 897 Hfq-dependent genes belong to more than 19 functional categories (Fig. 3 and Table S1 and S2). Several categories of genes were more notably affected by the absence of *hfq*. 34.3% (36/105) of the genes in the signal transduction category were deregulated in the Δ*hfq* strain, of which, 14 were up-regulated and 22 were down-regulated. In addition, Hfq-dependency accounts for 35.7%, 30.6%, 26.36%, and 18% of genes belonging to the categories of energy production and conversion, transport and metabolism of lipid, carbohydrate, and amino acid, respectively (Fig. 3).

### Hfq modulated the production of K2 capsular polysaccharides

**Figure 4:**
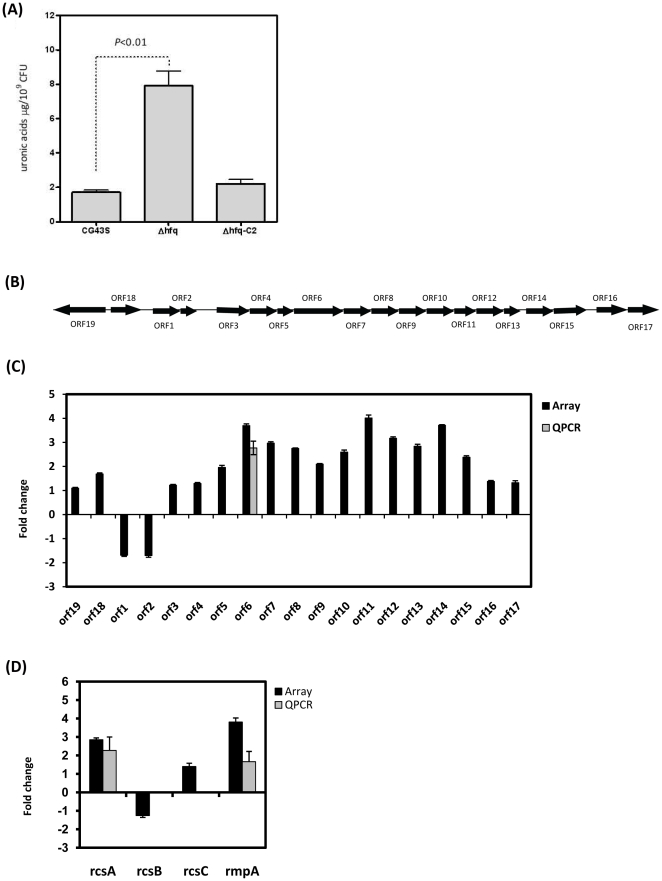
Hfq modulated the production and expression of K2 CPS. (**A**) Enhancement of K2 capsular polysaccharides by the deletion of *hfq*. Capsular polysaccharides were extracted from overnight-cultured *K. pneumoniae* CG43S, Δ*hfq*, and the complementation strain Δ*hfq*-C2 by the method described previously [56]. The amount of K2 CPS was reflected by the uronic acid content that was determined by the method described [57] from a glucuronic acid standard curve and expressed as micrograms per 10^9^ CFU. The indicated *P* values were determined using the Student’s *t*-test. (**B**) Genomic organization of K2 *cps* gene cluster which is depicted from that in *K. pneumoniae* Chedid (NCBI accession no. D21242.1). (**C**) Fold changes in transcript abundances of K2 *cps* genes detected by microarray (black bars) and QPCR (grey bars) in Δ*hfq* relative to that in CG43S are indicated. (**D**) Fold changes in transcript abundances of CPS-regulating genes, *rcsA*, *rcsB*, *rcsC*, and *rmpA* detected by microarray (black bars) and QPCR (grey bars) in Δ*hfq* relative to that in CG43S are indicated.

Capsule has been established as a virulence determinant in *K. pneumoniae*. Interestingly, Δ*hfq* failed to induce a systemic infection in mice but exhibited an enhanced hypermucoviscosity phenotype with 4.4-fold more K2-specific capsular polysaccharides (CPS) production compared to CG43S. The quantity of K2-CPS was restored to a normal level in Δ*hfq* by the introduction of *hfq*-complementing plasmid (Δ*hfq*-C2) (Fig. 4A). As revealed by the microarray analysis, abundance of the major transcript *orf3*-*orf15* of K2 *cps* gene cluster (D21242.1; Fig. 4B) increased in the range of 2–4 folds in the Δ*hfq* strain as compared to that in CG43S (Fig. 4C). Furthermore, K2 *cps* genes have been shown to be regulated by transcriptional activators, RcsA [28] and RmpA [29], whose transcripts both significantly increased in the absence of *hfq*. These results suggested that Hfq may modulate the production of K2 CPS by negatively regulate the expression of RmpA and RcsA at the post-transcriptional level.

### A loss of *hfq* altered expression profiles of envelope proteins

**Figure 5:**
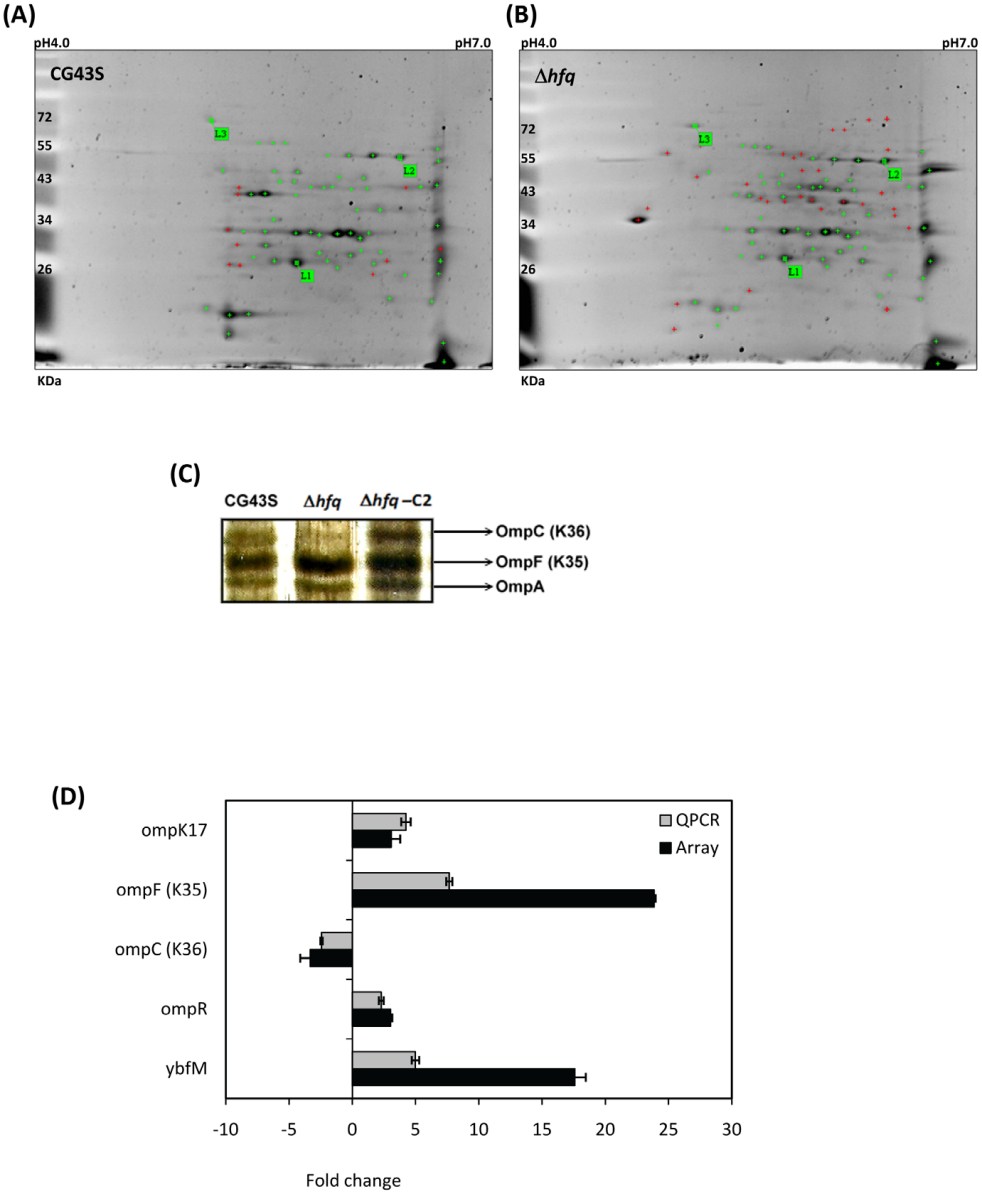
Hfq modulated the expression profiles of envelop proteins. Two hundred micrograms of extracytoplasmic proteins isolated from overnight-cultured *K. pneumoniae* CG43S (A) or Δ*hfq* (B) were electrophoresized with two-dimension gels. Images of silver-stained gels analyzed with ImageMasterTM 2D Platinum Version 5.0 are shown. L1-3: landmarks for comparison. Proteins whose expression levels are significantly higher in CG43S or Δ*hfq* are indicated with a red cross. (**C**) Outer membrane proteins were extracted, fractionated by 12% of SDS-PAGE gel, and silver-stained. The portion of the gel with bands corresponding to the major porins, OmpC, OmpF, and OmpA, is presented. (**D**) Fold changes in transcript abundances of *ompK17*, *ompF*, *ompC*, *ompR*, and *ybfM* detected by microarray (black bars) and QPCR (grey bars) in Δ*hfq* relative to that in CG43S are indicated.

Previous studies indicated that lacking *hfq* caused accumulation of outer membrane proteins and thus induced an envelope stress in UPEC, *Salmonella*, and *Vibrio* [16], [22], [23]. To examine whether a loss of *hfq* affected the expression profiles of *K. pneumoniae* envelope proteins, two-dimensional gel analysis was utilized to compare extracytoplasmic proteins which were purified from cultures of Δ*hfq* and CG43S grown aerobically in LB medium. As shown in Fig. 5A, a significant difference in the expression of extracytoplasmic proteins between Δ*hfq* and CG43S was noted. Among 94 proteins analyzed, 32 proteins were up-regulated and 10 were down-regulated in the absence of Hfq. Analyses of outer membrane proteins in one-dimensional gels revealed an increase in the protein levels of OmpF (OmpK36) and a decrease of OmpC (OmpK35) in Δ*hfq* as compared to those in CG43S and in the trans-complemented strain Δ*hfq*-C2 (Fig. 5B). These changes were consistent with the microarray data that the deletion of *hfq* caused the levels of *ompF* mRNA to rise by 23.9 fold and the *ompC* mRNA to drop by 3.3 fold (Fig. 5C).

### Δ*hfq* lost its stress tolerance to H_2_O_2_, heat shock, and UV radiation

**Figure 6:**
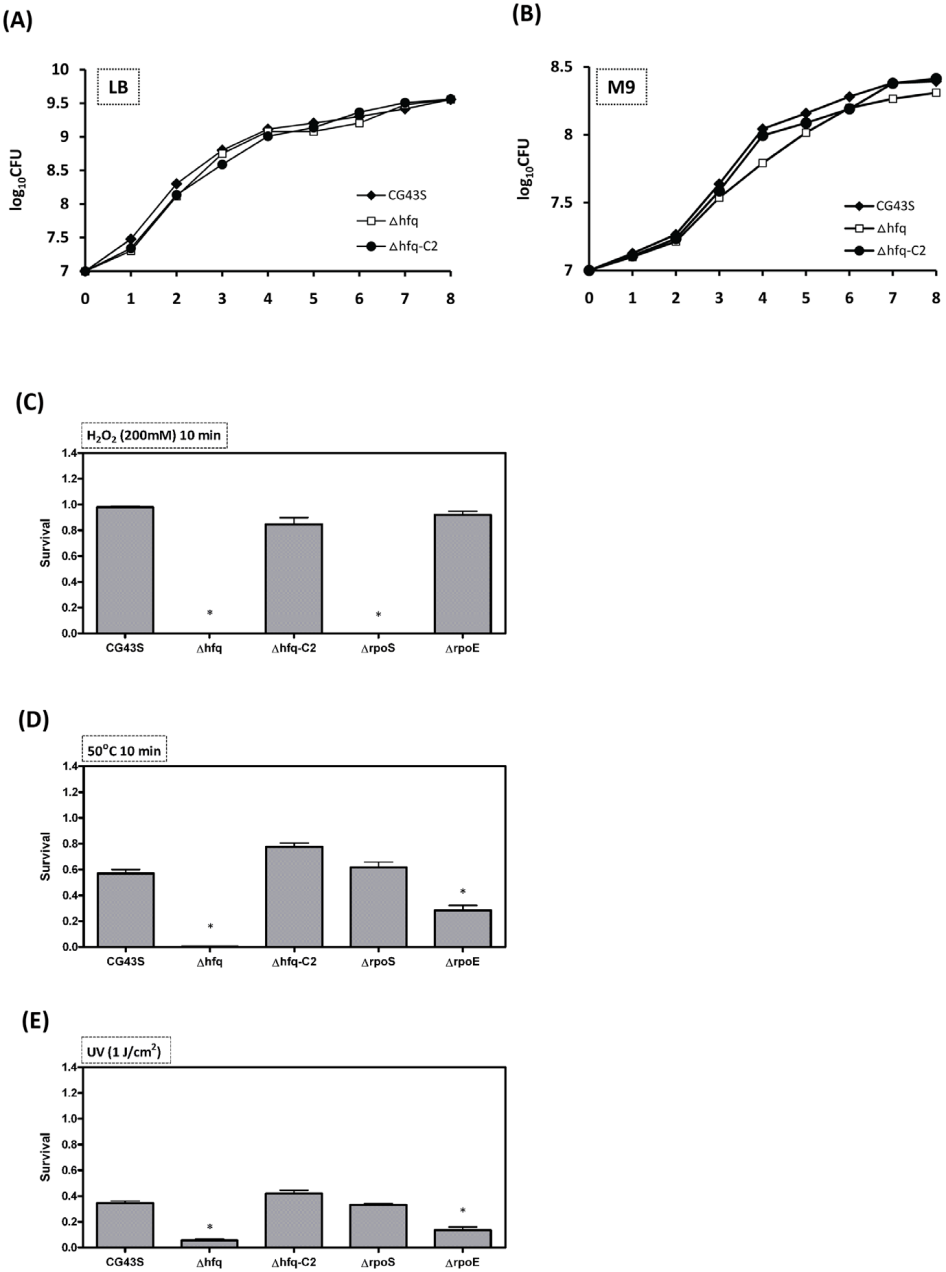
Stress tolerance attenuated in *K. pneumoniae* lacking *hfq*, *rpoS*, or *rpoE*. Growth of *K. pneumoniae* CG43S (filled diamonds), Δ*hfq* (open squares), and Δ*hfq*-C2 (filled circles) in (**A**) LB and (**B**) M9 media is determined by CFU calculation at indicative time points. Survival of *K. pneumoniae* CG43S, Δ*hfq*, Δ*hfq*-C2, Δ*rpoS*, and Δ*rpoE*, upon the treatment of 200 mM H_2_O_2_ for 10 minutes (**C**), 50°C shock for 10 minutes (**D**), or UV irradiation (1 J/cm^2^) (**E**) are determined by CFU calculation and presented as (CFU after treatment/CFU before treatment) ×100%. * *P*<0.05, determined with the Student’s *t*-test.

To determine whether Hfq was required for *K. pneumoniae* to cope with stresses that it may encounter inside the host, the growth of Δ*hfq* was characterized in various stressful conditions. As shown in Fig. 6, while Δ*hfq* grew normally in LB medium (Fig. 6A), the growth of Δ*hfq* lagged behind that of CG43S by approximately one hour and reached a lower density at saturation when the strains were grown in minimal medium (Fig. 6B), suggesting that the loss of Hfq caused only minor effects on *K. pneumoniae* response to nutrient deficiency. On the other hand, the loss of *hfq* rendered *K. pneumoniae* incapable of surviving after 10 minutes of treatment with H_2_O_2_ (Fig. 6C) or heat shock (Fig. 6D), and also increased the sensitivity of *K. pneumoniae* to ultraviolet (UV) irradiation (Fig. 6E). The reduced tolerance of Δ*hfq* to the stresses was restored to the wild-type level by the introduction of the *hfq*-complementing plasmid driven by its native promoters (Δ*hfq*-C2) (Fig. 6C, D, and E).

### Overlap among Hfq-, RpoS-, and RpoE-regulons

**Figure 7:**
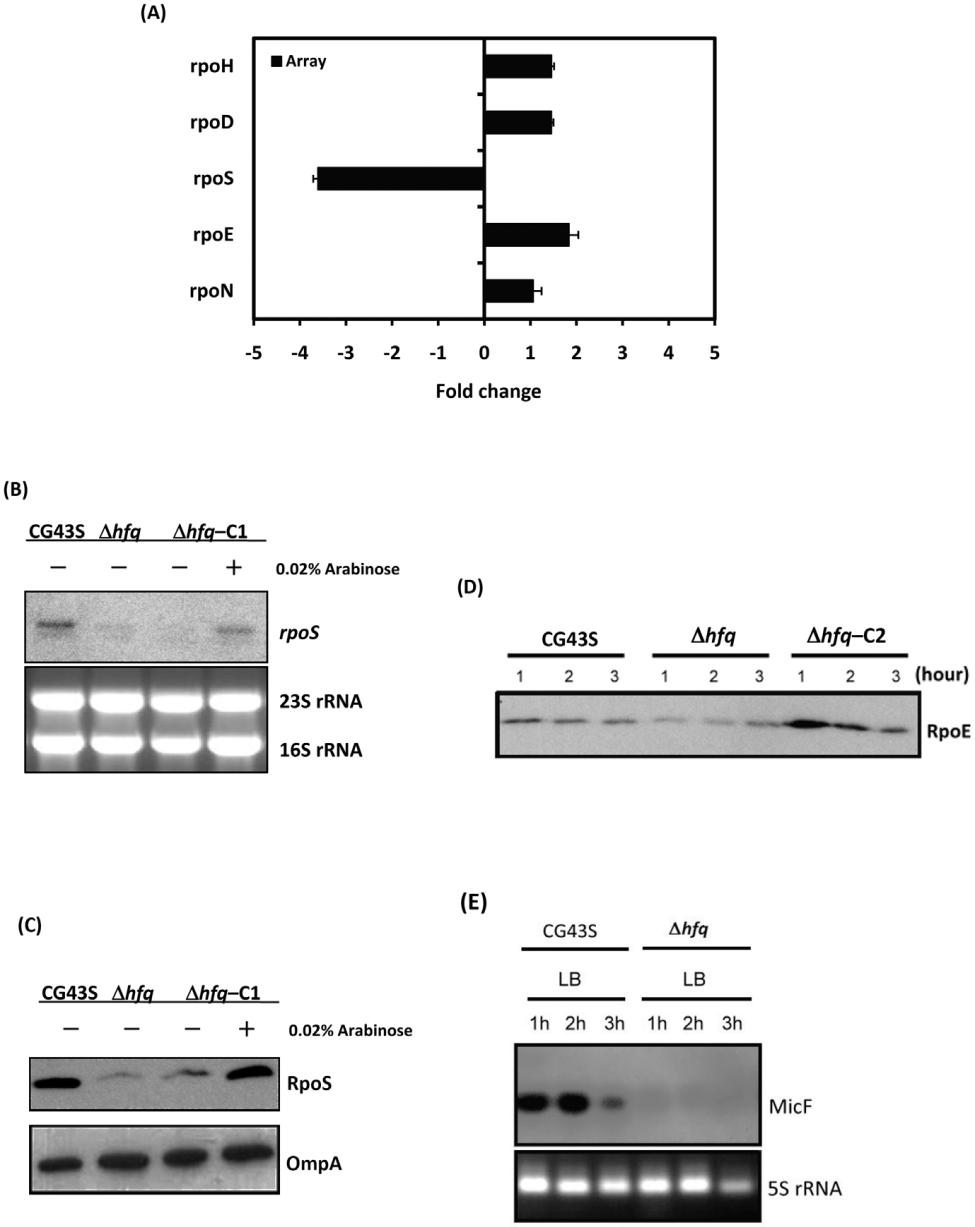
Hfq affected the expression of sigma factors and small RNAs. (**A**) Fold changes in transcript abundances of *rpoH, rpoD, rpoS, rpoE,* and *rpoN* detected by microarray (black bars) in Δ*hfq* relative to that in CG43S are indicated. (**B**) Transcripts of *rpoS* extracted from CG43S, Δ*hfq*, and Δ*hfq*-C1 were detected by Northern blotting with an *rpoS*-specific biotin-labeled riboprobe. (**C**) RpoS proteins isolated from the LB-grown stationary-phase cultures of CG43S, Δ*hfq*, and Δ*hfq*-C2 with or without the induction of 0.02% arabinose were detected by Western blotting with rabbit anti-RpoS antibody. The levels of OmpA were detected with rabbit anti-OmpA antibody as a loading control. (**D**) RpoE proteins isolated from the LB-grown cultures of CG43S, Δ*hfq*, and Δ*hfq*-C1 at indicated time points were detected by Western blotting with rabbit anti-RpoE antibody. (**E**) Transcripts of MicF extracted from the LB-grown cultures of CG43S and Δ*hfq* at indicated time points were detected by Northern blotting with a MicF-specific biotin-labeled riboprobe.

Our microarray data showed that the transcript level of *rpoS* was down-regulated by 3.6-fold in the absence of *hfq* (Fig. 7A). The decrease of *rpoS* transcripts in Δ*hfq* was restored to the wild-type level by the complementation of *hfq* under the control of pBAD promoter and the restoration was confirmed by Northern blotting analysis (Fig. 7B). In accordance with this, the protein level of RpoS was down-regulated by the absence of Hfq (Fig. 7C). To determine whether the virulence attenuation and phenotypic alterations observed in the Δ*hfq* strain was attributed to the downregulation of *rpoS* by the loss of Hfq, an *rpoS* deletion mutant (Δ*rpoS*) was generated in *K. pneumoniae* CG43S. Unlike Δ*hfq*, whose virulence to mice was significantly attenuated, Δ*rpoS* behaved much like CG43S as it caused 80% mortality of mice within one week (open diamonds, Fig. 2B). Δ*rpoS* also displayed wild-type-level tolerance of *K. pneumoniae* in response to heat shock and UV irradiation (Fig. 6D and 6E). On the other hand, the loss of *rpoS* did abolish the ability of *K. pneumoniae* to conquer H_2_O_2_ stress (Fig. 6C). The results suggested that the downregulation of *rpoS* in the Δ*hfq* strain contributed partially to the defects on stress tolerance resulted from the loss of *hfq*, but could not by itself attenuate the virulence of *K. pneumoniae*. Meanwhile, the expression of RpoE had also been examined. Although the transcript level of *rpoE* in the Δ*hfq* strain was found similar to that in CG43S in the microarray analysis, Western blotting analysis revealed that the absence of *hfq* resulted in decreased protein level of RpoE at early- and mid-log phase (Fig. 7C). An *rpoE* deletion mutant (Δ*rpoE*) was generated. Unlike Δ*rpoS*, Δ*rpoE* was totally avirulent when given intraperitoneally with the same inoculums that caused 80% mortality in the Δ*rpoS*-infected group (open diamonds, Fig. 3B). Δ*rpoE* was as sensitive as Δ*hfq* in its responses to heat shock and UV irradiation (Fig. 6D and 6E), whereas it exhibited a wild-type-level resistance to H_2_O_2_ (Fig. 6C). These results suggested that the virulence attenuation as well as the loss of tolerance to heat shock and UV irradiation in Δ*hfq* may result from the decrease of RpoE protein by the lack of *hfq*.

To further determine whether the set of Hfq-dependent genes overlapped with those belonging to the RpoS- and/or RpoE-regulon, genes whose transcripts were deregulated significantly upon the overexpression of RpoS or RpoE were identified. As revealed by microarray analyses, among the 610 down-regulated genes identified in Δ*hfq*, the transcript abundance of 90 and 109 genes were significantly affected by overexpression of RpoE and RpoS (Table S1), while 85 and 46 genes out of the 287 up-regulated genes were under control of RpoE and RpoS, respectively (Table S2). Overall, 19.5% (175/897) and 17.3% (155/897) of Hfq-dependent genes belong to the RpoE- and RpoS-regulon, respectively, and 4.8% (43/897) of Hfq-dependent genes were presented in both regulons. These results suggested that the Hfq-mediated downregulation of RpoS and RpoE was responsible for the Hfq-dependency for 32% of the 897 genes identified in the Δ*hfq* strain.

### Hfq-dependent changes in transcript abundances of sRNAs in *K. pneumoniae*

As an RNA chaperone which is critical in stabilizing sRNA-mRNA complexes, Hfq is required for the trans-acting sRNAs to regulate the expression of their target mRNAs [30]. Most of the Hfq-dependent sRNAs are less stable and no longer regulate their target mRNAs in *E. coli* lacking *hfq* [31]. Therefore, it was likely that the abundance of a number of Hfq-dependent genes were affected by the change of sRNA abundance in the absence of Hfq. To test this possibility, 33 *K. pneumoniae* sRNA genes which have homologues in *E. coli* K-12 were identified from the genome of *K. pneumoniae* NTHU-K2044 (Table 2) and their transcripts abundances was examined by microarray analyses. As shown in Table 2, 25 *K. pneumoniae* sRNAs were downregulated by the absence of Hfq, of which, 11 sRNAs (GcvB, MicF, RyeB, RyeE, RygB, RyhA, RyiA, SraB, MicM, SroC, and SroG) exhibited >3-fold lower transcript abundance in Δ*hfq* than in CG43S. Among them, the diminishment in transcript abundance of MicF was confirmed by Northern blotting (Fig. 7D). In accordance with the finding in *E. coli* that MicF inhibited the translation of *ompF* mRNA [4], the decline of MicF in the Δ*hfq* strain derepressed the negative control of *ompF* transcript and consequently enhanced the expression of *K. pneumoniae* OmpF protein (Fig. 7A and 7B). The identification of sRNAs that required Hfq for maintaining their abundance in *K. pneumoniae* suggested that a subset of *K. pneumoniae* genes whose expression changed in the absence of Hfq were controlled by the Hfq-dependent sRNAs.

**Table 2:** Hfq-dependent changes in transcript abundances of sRNAs in *K. pneumoniae*.

No.	Homologues in *E. coli*	Alternative name	Length (bp)	Fold change bythe absence of *hfq* a
**Sr0006**	DsrA		85	−2.20±0.27
**Sr0008**	GcvB		207	**−8.40±0.06**
**Sr0009**	IstR-1		76	−1.48±0.06
**Sr0011**	MicC		112	1.03±0.12
**Sr0012**	MicF		93	**−9.45±0.04**
**Sr0013**	OxyS		109	−1.74±0.21
**Sr0015**	RprA		105	−2.57±0.11
**Sr0017**	RybA		89	2.36±0.10
**Sr0018**	RybB		80	−1.39±0.16
**Sr0019**	RydB		68	−2.69±0.12
**Sr0020**	RyeA	SraC	253	3.07±0.15
**Sr0021**	RyeB		124	**−9.71±0.07**
**Sr0022**	RyeE	CyaR	84	**−14.52±0.09**
**Sr0023**	RyfA		338	−1.10±0.15
**Sr0024**	RygA	OmrA	88	−1.16±0.06
**Sr0025**	RygB	OmrB	76	**−5.21±0.11**
**Sr0026**	RyhA	SraH, ArcZ	115	**−16.11±0.12**
**Sr0027**	RyhB	RhyB	96	−1.22±0.09
**Sr0028**	RyiA	GlmZ	177	**−3.47±0.06**
**Sr0029**	RyjA		147	−2.18±0.04
**Sr0030**	SgrS	RyaA	242	−2.11±0.13
**Sr0032**	SraA		158	−1.02±0.21
**Sr0033**	SraB		173	**−4.47±0.12**
**Sr0034**	MicA		77	2.51±0.09
**Sr0035**	SraF		188	2.41±0.04
**Sr0036**	SraG		167	1.22±0.58
**Sr0037**	SroA		100	1.85±0.05
**Sr0038**	MicM	SroB, RybC	84	**−6.36±0.13**
**Sr0039**	SroC		159	**−4.56±0.09**
**Sr0040**	SroD		87	1.27±0.15
**Sr0041**	SroE		97	−1.75±0.08
**Sr0042**	SroF		183	−1.93±0.08
**Sr0043**	SroG		152	**−6.02±0.16**

Note. The transcript abundance of 33 *K. pneumoniae* sRNAs was determined by microarray analysis as described in Materials and Methods.

a Fold change by the absence of *hfq* represents the transcript abundance in Δ*hfq* compared to that in CG43S. Positive numbers indicate increases; negative numbers indicate decreases. Bold represents >3-fold change.

## Discussion

Small RNAs, along with Hfq, are emerging as regulators that enable bacteria to modulate gene expression in response to changing environments. In this study, we demonstrate that Hfq modulates the expressions of a wide range of genes and thus regulates the physiology and virulence of *K. pneumoniae*. In the absence of Hfq, *K. pneumoniae* drastically lost its ability to disseminate into extra-intestinal organs, and was unable to induce a systemic infection. Comparison of the Δ*hfq* strain with its parental strain revealed that several physiological characteristics were altered due to the Hfq deficiency, including production of capsular polysaccharides, envelope protein expression profiles, and tolerance to H_2_O_2_, heat shock, and UV irradiation. Genome-wide transcriptome analyses showed that 897 genes involved in numerous cellular processes had >2.83-fold change in their transcript abundance in Δ*hfq*. The apparent influence of Hfq on almost a fifth of *K. pneumoniae* genes indicated that Hfq acted as a global regulator. Since RpoS and RpoE were found to be down-regulated in Δ*hfq*, Hfq may also govern gene expression indirectly in *K. pneumoniae* through its positive effect on the availability of sigma factors. The idea was supported by the microarray data that 32% of Hfq-dependent genes belong to the RpoE- and/or RpoS-regulon. Moreover, reduction on the transcript levels of 25 *K. pneumoniae* sRNAs (out of 33 known sRNAs) in Δ*hfq* indicated that a large proportion of genes that were affected in the absence of Hfq were mediated through the action of sRNAs.

**Figure 8:**
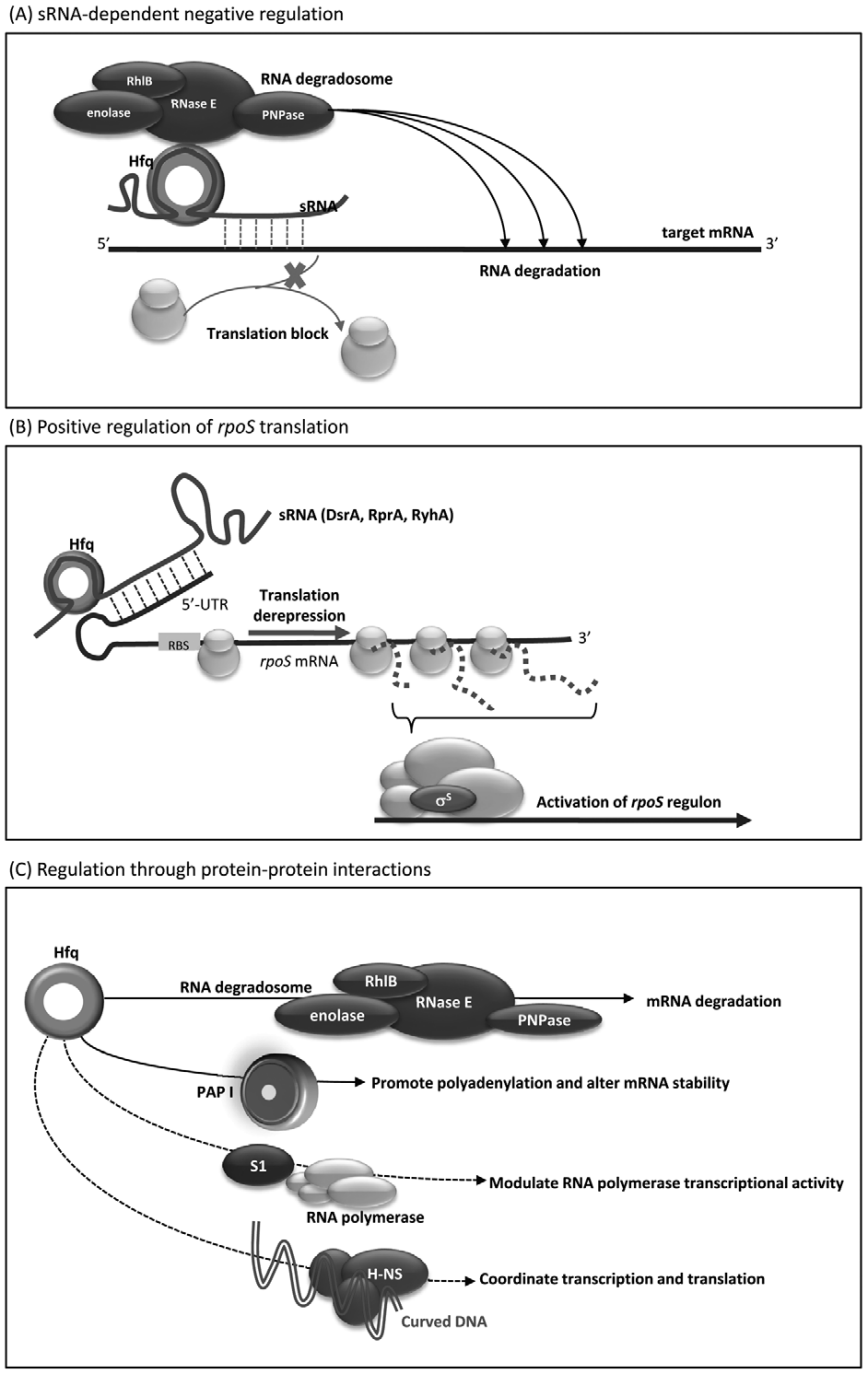
Schematic representation of possible mechanisms of Hfq-mediated regulation. (**A**) **sRNA-dependent negative regulation.** Hfq-dependent sRNAs bind their target mRNAs with partial complementarity that occlude the ribosome binding sites, prevents ribosome association and thus represses translation. In many cases, translation inhibition can be coupled to RNA degradation. Hfq recruits RNA degradosome and RNA degradation will be achieved at distal sites from the paring region. (**B**) **Positive regulation of** ***rpoS*** **translation.** Hfq-dependent sRNAs, such as DsrA, RprA, and RyhA, relieve an inhibitory secondary structure formed by the *rpoS* mRNA leader sequence. Derepression of *rpoS* translation increases the availability of RpoS (σ^S^) and thus activates the expression of genes belonging to the *rpoS* regulon. (**C**) **Regulation through protein-protein interactions.** Hfq may contact with proteins to affect cellular processes. Hfq can recruit RNA degradosome, consisting of RNaseE, RhlB helicase, enolase, and PNPase, to stimulate the RNA degradation of sRNA-mRNA duplex. Hfq may alter mRNA stability by promoting PAP I-mediated polyadenylation. In the presence of S1 protein, Hfq may interact with RNA polymerase to modulate transcriptional activities. A minor fraction of Hfq, which was found to associate with the nucleoid, may bind curved DNA, affect negative supercoiling, and then coordinate the transcription and translation activities.

The majority of the known sRNA-mediated regulation is negative [8], [30]. As shown in Fig. 8A, with the aid of Hfq, sRNAs bind to the 5’-UTR of a single or multiple target mRNAs, occlude the ribosome-binding site, prevent ribosome association, and thus inhibit translation initiation. In many cases, the sRNA-mRNA duplex is then subject to degradation [32]. Hfq recruits the RNA degradosome, consisting of RNase E, PNPase, RhlB helicase, and enolase, and RNA degradation is triggered by the RNase E cleavage at sites distal from the pairing region [11]. Based on this model, we speculate that the absence of Hfq relieves the sRNAs-mediated negative regulation and elevates the transcript abundances of genes that are targeted by certain sRNAs. In line with this assumption, a significant number of 287 up-regulated genes identified in Δ*hfq* (Table S2) might be subject to this kind of Hfq-sRNA-mediated negative regulation in *K. pneumoniae*. On the other hand, unlike eukaryotic miRNAs, there are three bacterial sRNAs, DsrA, RprA, and RyhA, have been identified to activate the expression of *rpoS* through anti-antisense mechanism whereby basepairing of the sRNAs disrupts an inhibitory secondary structure formed by the *rpoS* mRNA leader sequence [30], [33]. As shown in Fig. 8B, derepression of *rpoS* translation increases the availability of RpoS (σ^S^), and activates the expression of genes belonging to the *rpoS* regulon. Therefore, due to a loss of positive regulation by sRNAs, the absence of Hfq caused the reduced translation efficiency of *rpoS* (Fig. 7A–C). This result is consistent with some of the changes in the gene expression profile observed in Δ*hfq*, as shown by microarray data that 17.3% of Hfq-dependent genes fall in the RpoS regulon. In addition to the influence on translational efficiency of *rpoS*, the decreased transcript level of *rpoS* suggested that Hfq-dependent regulation of *rpoS* in *K. pneumoniae* was more complicated than that in *E. coli* and *Salmonella* [30], [33]. Although the mechanism requires further studies to clarify, the existence of Hfq-dependent transcriptional activators of *rpoS* or the possibility that a loss of *hfq* affects the stability of *rpoS* transcript may explain this result.

Hfq acts as a global regulator in a variety of pathogens [9], however, the spectrum and severity of mutant phenotypes observed upon the deletion of *hfq* varied among the different pathogens so far analyzed. In *S.* Typhimurium and *E. coli* [33], [34], the involvement of Hfq in bacterial virulence was indicated by its requirement for translation derepression of RpoS. In *K. pneumoniae*, although the expression of *rpoS* (Fig. 7A and 7B) and the RpoS-mediated oxidative stress response (Fig. 6C) was affected by the absence of Hfq, Δ*hfq* was far more attenuated in causing a systemic infection than was Δ*rpoS* (Fig. 2B). The dispensability of *rpoS* for *K. pneumoniae* virulence suggests that the Hfq-dependent control of virulence genes in *K. pneumoniae* is largely mediated through an RpoS-independent manner, which is in accordance with previous studies that the virulence defect of *hfq* mutants in *V. cholera* and *B. abortus* is independent of the effects on RpoS expression [14], [16], [35].

In addition to RpoS, the expression of envelope stress sigma factor, RpoE, was also affected by the absence of Hfq. While Δ*hfq* lost its capacity to tolerate oxidative stress as Δ*rpoS* (Fig. 6C), the increased sensitivity to heat shock and UV irradiation in the absence of Hfq more closely resembled the phenotypes observed in Δ*rpoE* (Fig. 6D and 6E). The Hfq-associated regulation on RpoE expression varied among different bacteria. Previous studies indicated that Hfq negatively regulated the expression of RpoE in *E. coli* [36], *S.* Typhimurium [22], and *V. cholerae* [16]. However, in *Rhodobacter sphaeroides* [37] and *Sinorhizobium meliloti* [38], Hfq exerted a positive effect on RpoE. Interestingly, in *K. pneumoniae*, despite the transcript abundance of *rpoE* was slightly increased in the absence of Hfq (Fig. 7A), the protein level of RpoE was decreased in the Δ*hfq* strain and was elevated by the introduction of an Hfq-complementation plasmid (Fig. 7C). The result indicated that the expression of RpoE might be regulated by Hfq at multiple levels. The deletion of *hfq* in *K. pneumoniae* caused modification of the expression profiles of OMPs. As shown in the proteomic result, 10 extracellular proteins were down-regulated and 32 proteins were up-regulated (Fig. 5A and 5B). The disturbance of envelope homeostasis in Δ*hfq* could induce the envelope stress and release RpoE from the anti-sigma factor RseA [36]. Upon the overexpression of RpoE, as revealed by microarray data, the transcriptional level of *rpoE*-*rseA*-*rseB* operon and RybB were respectively elevated to >9-fold and 300-fold over the vector control, suggesting that the increased anti-sigma factor RseA and RseB as well as the RpoE-inhibiting sRNA, RybB, might exert a feedback control by down-regulating the elevated level of RpoE protein at either post-transcriptional or post-translational level. The decrease of RpoE protein in Δ*hfq* might be an outcome of the net effect from the induction of RpoE by the envelope stress and the subsequent feedback control of RpoE by its negative regulators, RseA, RseB, and RybB. However, as the *rybB* transcript abundance was slightly reduced in Δ*hfq* with a fold change of -1.39 (Table 2), there might be some other Hfq-dependent factors involved in the negative regulation of RpoE in *K. pneumoniae*. Although further studies are needed to unveil the underlying mechanism, the decreased RpoE level in Δ*hfq* can partially explain the overlap of stress responsiveness and virulence attenuation observed among Δ*hfq* and Δ*rpoE* (Fig. 2B).

In our oral infection mouse model [39], *K. pneumoniae* lacking *hfq* retained its intestine-colonization ability but failed to disseminate into extra-intestinal tissues that attenuated the induction of a systemic infection (Fig. 1). Why did not the loss of Hfq abrogate the ability of *K. pneumoniae* to establish intestinal colonization? Transcripts abundances of genes belonging to the type III fimbriae-encoding operon were significantly up-regulated in the absence of Hfq (Table S2). As demonstrated in our previous study that the type III fimbriae was a major factor for *K. pneumoniae* to establish its intestinal colonization [39], the increased production of type III fimbriae in Δ*hfq* may compensate for the possible defects on intestinal colonization caused by the deletion of Hfq and can therefore render Δ*hfq* with the wild-type-level colonizing ability. As the overproduction of RpoS caused 3-10 fold decrease in the transcripts abundances of type III fimbriae-encoding genes, the Hfq-dependent control on type III fimbriae expression might be attributed to the derepression of *rpoS* in Δ*hfq* (Table S2). On the other hand, since capsule had been established as a virulence determinant for *K. pneumoniae* pathogenesis, it was interesting that Δ*hfq* produced more K2 capsular polysaccharides, but failed to develop a systemic infection. It seems that the enhancement of capsular polysaccharides may not be advantageous in all cases. As our microarray data revealed that almost a fifth of genes (123/516; 23.8%) of the carbohydrate transport and metabolism category were significantly downregulated in Δ*hfq* (Fig. 3), the overproduction of capsular polysaccharides in the absence of Hfq may unbalance the metabolic flow of carbon sources and therefore affects the bacterial physiology under a nutrient-deprivation surrounding, such as inside the host during infections. Since *K. pneumoniae* can survive upon different circumstances, according to the result, we speculate that Hfq may modulate the expression of capsular polysaccharides to set a balance between the carbon usage and the production of capsule, which benefits this bacterium with an adaptive potential to switch among a panel of growth programs.

Most sRNAs have negative impacts on gene expression in bacteria. Genes whose expression is subject to the control of sRNAs are believed to be up-regulated by the loss of *hfq*. Interestingly, in our study, the number of genes that were down-regulated (n = 610) was more than twice the number of up-regulated genes (n = 287) in Δ*hfq K. pneumoniae.* The positive effects of Hfq on gene expression in *K. pneumoniae* may be mediated through upregulation of sigma factors, or repression of sRNA-controlled transcriptional repressors, or through an sRNA-independent manner. It has been shown that Hfq per se can bind with mRNAs, tRNAs [40], and various proteins. In addition to direct interacting with Qβ phage RNA [41], Hfq binds its own mRNA that inhibits the formation of translational initiation complex to autoregulate its own expression [42]. A variety of proteins, including ribosomal proteins, RNases, helicases, Rho-factor, RNA polymerase, protein H-NS, polynucleotide phosphorylase (PNPase), and poly(A)polymerase (PAP I), exhibited a direct interaction with Hfq [32], [43], [44], [45], [46]. We speculate that Hfq can contact with proteins to influence *K. pneumoniae* gene expression. As shown in Fig. 8C, Hfq can recruit the RNA degradosome to stimulate RNA degradation. Hfq can alter mRNA stability by promoting PAP I-mediated polyadenylation [47], [48], [49]. In the presence of S1 protein, Hfq may interact with RNA polymerase to modulate transcriptional activities [46]. Although the majority of Hfq is located in the cytoplasm, it has been found that a minor fraction of Hfq associates with the nucleoid that preferentially binds curved DNA. This observation suggests that with the dual binding capacity to both DNA and RNA, Hfq may have a role in functional coordination between transcription and translation [50]. Taken together, the impact of Hfq on global gene expression that controls the physiological fitness and virulence potential of *K. pneumoniae* shown by this work emphasizes the role of Hfq as a pivotal coordinator for integrating a diversity of regulatory circuits and also the potential that Hfq may serve as a scaffold molecule for the design of novel antimicrobial drugs.

## Materials and Methods

### Ethics Statement

All animal experiments were performed in strict accordance with the recommendation in the Guide for the Care and Use of Laboratory Animals of the National Laboratory Animal Center (Taiwan), and the protocol was approved by the Animal Experimental Center of Chung-Shan Medical University (Permit number: 694). All surgery was performed under anesthesia, and all efforts were made to minimize suffering.

### Strains, plasmids, and primers

Bacterial strains, plasmids, and primers used in this study are listed in Table 1. *E. coli* and *K. pneumoniae* CG43 and its derivatives were propagated in Luria-Bertani (LB) broth. Gene-specific deletion mutants were generated with an allelic exchange technique as described previously [51]. In general, approximately 1,200-bp DNA fragments flanking the coding region of genes to be deleted (*hfq* or *rpoE*) were amplified with specific primer sets, p239/p240 and p241/p242 for *hfq* deletion; p251/p252 and p253/p254 for *rpoE* deletion and cloned into the suicide vector, pKAS46 [52]. To facilitate the positive selection of transconjugants, a chloramphenicol-resistant or a tetracycline-resistant cassette which was amplified from pACYC184, cloned into the inserts on pKAS46 constructs to replace the deletion region, and the resulting plasmids for homologous recombination of *hfq* and *rpoE* were pYC324 (Cm^r^) and pYC445 (Tc^r^), respectively. After the occurrence of double crossover, the chloramphenicol-resistant or tetracycline-resistant colonies were selected, and the deletions of *hfq* or *rpoE* were verified by PCR and Southern blot analysis. The *rpoS* deletion mutant (Δ*rpoS*) which was generated as described [53] was kindly provided by Dr. Lin, G.T. (China Medical University, Taiwan).

**Table 1:** Strains, plasmids, and primers used in this study.

	Description	Source
**Strains**		
***K. pneumoniae***		
**CG43**	Wild type bacteremia isolates	[58]
**CG43S**	CG43 Sm^r^	[25]
**Δ** ***hfq***	CG43S Δ*hfq*::Cm^r^	This study
**Δ** ***hfq*** ** -C1**	Δ*hfq* complemented with pYC343	This study
**Δ** ***hfq*** ** -C2**	Δ*hfq* complemented with pYC457	This study
**Δ** ***rpoS***	CG43S Δ*rpoS*	C.T. Lin
**Δ** ***rpoE***	CG43S Δ*rpoE*::Tc^r^	This study
***E. coli***		
**S17-1λ** ***pir***	*hsdR recA pro* RP4-2 (Tc::Mu; Kan::Tn*7*)	[52]
**TOP10**	F^−^ *mcrA* Δ(*mrr-hsdRMS-mcrBC*) *lac*ZΔM15 *rpsL* (Sm^r^) *end*A1	Invitrogen
**Plasmids**		
**pKAS46**	Homologous recombination vector, *rpsL* couterselection	[52]
**pBAD-202**	Arabinose-inducible TOPO expression vector	Invitrogen
**pACYC184**	Low-copy-no. vector containing Ap^r^ and Kan^r^ cassettes	New England Biolabs
**pYC324**	2.4-kb fragment containing a 309-bp deletion in *hfq* locus cloned into pKAS46	This study
**pYC343**	*hfq* coding region cloned from CG43 into pBAD-202	This study
**pYC351**	*rpoS* coding region cloned from CG43 into pBAD-202	This study
**pYC413**	*rpoE* coding region cloned from CG43 into pBAD-202	This study
**pYC457**	Full length *hfq* with native promoter ligated into pACYC184 backbone	This study
**pYC445**	2.4-kb fragment containing a 609-bp deletion in *rpoE* locus cloned into pKAS46	This study
**Primers**		This study
**p239**	TgCTCTAgATTCCTgAACTgATAggCT	This study
**p240**	CggggTACCTgTACACgTTCAgTTCTgg	This study
**p241**	CggggTACCTgTCTCACCACAgCAACA	This study
**p242**	gCCgAgCTCTTTCATCgTCgCgATCgA	This study
**P403**	ggCgAATTCCgCTgTTAgTC	This study
**p284**	CACCATggCTAAggggCAATCT	This study
**p285**	TTCggCgTCgTCgCTgTC	This study
**p251**	TAgCTATAgTTCTAgAgCTTCATTTCATggTCgA	This study
**p252**	CgCCAgCTgCAggCggCCgCCTgCATTATgAgCAAgCTg	This study
**p253**	AggATgCATATggCggCCgCTgACgATAgCggAATACTg	This study
**p254**	TgTggAATTCCCgggAgAgCTCTgACgTTATCgCCAACg	This study
**p903**	CACCATgAgCgAgCAgTTAACg	This study
**p904**	ACgCCTgATAAgCggTTg	This study
**p909**	CACCATgAgTCAgAATACgCTg	This study
**p910**	TTCgCggAAgAgCgCTTC	This study

The entire 309 bp *hfq* coding sequence and 745 bp of upstream sequence were amplified from CG43 chromosomal DNA with primer set p403/p285 and cloned into pACYC184 to generate pYC457. The coding sequences of *hfq*, *rpoS*, and *rpoE* were amplified with primer sets, p284/p285, p903/p904, and p909/p910, respectively, and cloned into pBAD202 (Invitrogen) to generate pYC343, pYC351, and pYC413. The protein of Hfq, RpoS, or RpoE with a C-terminally fused His_6_-tag was induced by addition of 0.02% arabinose and verified using Western blot analysis with anti-His antibodies (Santa-Cruz). All these constructs were introduced into *K. pneumoniae* CG43S and its mutants through electroporation.

### Mouse infections

Eight-week-old male BALB/c mice (National Laboratory Animal Center, Taiwan) were injected intraperitoneally with 100 µl of bacterial suspension containing 10^4^ or 2×10^3^ CFU of mid-log *K. pneumoniae* CG43S or mutants. The survival rate of the infected mice was monitored daily for 2 weeks. Mortality rate and mean number of days to death (MDD) were determined by Kaplan-Meier analysis using Prism4 for Windows (GraphPad); *P* values of <0.05 were considered statistically significant. To further examine virulence attenuation of the Δ*hfq* strain, a competitive assay was performed as described previously [54]. One hundred microliters of bacterial suspension containing equal amounts of *K. pneumoniae* CG43S and Δ*hfq* was used to infect 8-week-old male BALB/c mice through an oral or an intraperitoneal route with inoculums of 1×10^9^ or 2×10^3^ CFU, respectively. At indicative time points after inoculation, viable counts of CG43S and Δ*hfq* in a particular mouse tissue were determined using M9 agar supplemented with or without chloramphenicol (10 µg/ml). Competitive index (CI) values were calculated as described [54].

### Microarray construction

*K. pneumoniae* microarray was customized using Agilent eArray 5.0 program according to the manufacturer’s recommendations. The customized microarray (4×44K) contained spots in eight duplicates with 5,076 gene-specific oligonucleotides (45–60 mers in length) representing 5,024 genes in *K. pneumoniae* NTHU-K2044 genome (NCBI accession no. NC_012731)[24], 33 sRNA-coding candidates in *K. pneumoniae* (as predicted by homologues of *E. coli* K12), and 19 K2 *cps* genes (NCBI accession no. D21242.1).

### RNA isolation, labeling, hybridization, and scanning

Total RNA was isolated from 20 ml of log *K. pneumoniae* culture by using Tri-reagent (Molecular Research Center), and purified by QIAGEN RNeasy cleanup kit. The total RNA yield was quantified by nanodrop UV spectroscopy (Ocean Optics) and the quantity was verified by gel electrophoresis and analyzed on a RNA 6000 Nano LabChip (Agilent Technologies) using a 2100 bioanalyzer (Agilent Technologies). cDNA was synthesized from 1 µg of enriched mRNA with Cyscribe 1^st^-strand cDNA labeling kit, (GE Healthcare) and labeled with Cy3 or Cy5 (CyDye, PerkinElmer). The fluorescently labeled cDNA was purified with QIAGEN RNeasy cleanup kit and fragmented in fragmentation buffer (Agilent). The correspondingly labeled cDNA was mixed in GEx Hybridization Buffer HI-RPM (Agilent). Hybridization was performed in an Agilent microarray Hybridization Chamber for 17 h at 60°C. After hybridization, the slides were washed in Gene Expression Wash Buffer (Agilent) and dried by nitrogen gun blowing. Microarrays were scanned using an Agilent microarray scanner at 535 nm for Cy3 and 625 nm for Cy5. Feature extraction 9.5.3 and image analysis software (Agilent Technologies) was used to locate and delineate every spot in the array, to integrate each spot’s intensity, and to normalize data using the rank-consistency-filtering Lowess method. The data points which had flag value of non-zero or a signal-to-noise ratio smaller than 2.6 were masked. The remaining data were log_2_ transformed and averaged for each gene. All microarray data reported in the study is described in accordance with MIAME guidelines and has been deposited in NCBI's Gene Expression Omnibus (GSE 29448). For selection of genes which were significantly regulated by Hfq, RpoE, or RpoS, a 1.5×log_2_ fold change (approximately 2.83-fold) and pValueLogRatio <0.05 were used as thresholds.

### Primer design and Real-time PCR

To prepare a cDNA pool from each RNA sample, total RNA (5 µg) was reverse transcribed using MMLV reverse transcriptase (Promega). Each cDNA pool was stored at −20°C until further real-time PCR analysis. Specific oligonucleotide primer pairs were selected from Roche Universal ProbeLibrary for real-time PCR assays. The specificity of each primer pair was validated by performing a RT-PCR reaction using pooled *K. pneumoniae* CG43S cDNA template, and the size of the PCR product was checked by a DNA 1000 chip (Agilent Technologies) run on Bioanalyzer 2100 (Agilent Technologies). Primer pairs of generating predicted product size and no other side-product were chosen to conduct the following real-time RT-PCR reaction.

Real-time PCR reactions were performed on the Roche LightCycler Instrument 1.5 using LightCycler&reg FastStart DNA MasterPLUS SYBR Green I kit (Roche). Briefly, 10 µl reactions contained 2 µl Master Mix, 2 µl of 3.75 µM or 2 µl of 2.5 µM with 5% DMSO, forward primer and reversed primer, and 6 µl cDNA sample. Each sample was run in triplicate. The RT-PCR program were 95°C for 10 min, 50 cycles of 95°C for 10 sec, 60°C for 15 sec, and 72°C for 10 sec. At the end of the program a melt curve analysis was done. At the end of each RT-PCR run, the data were automatically analyzed by the system and an amplification plot was generated for each cDNA sample. From each of these plots, the LightCycler3 Data analysis software automatically calculates CP value (crossing point, the turning point corresponds to the first maximum of the second derivative curve), which imply as the beginning of exponential amplification. The fold expression or repression of the target gene relative to internal control gene *rfaH* in each sample was calculated by the formula: 2^−△△CP^ where △Cp  =  Cp target gene – Cp internal control and △△Cp  =  △Cp test sample - △Cp control sample.

### Two-dimensional (2D) proteomic analysis

Extracytoplasmic proteins were extracted as described [55] with modifications. Briefly, 40 ml of *K. pneumoniae* CG43S or Δ*hfq* cultures were harvested at late-log phase by centrifugation at 13,000 rpm for 10 min at 4°C. The bacterial pellets were lysed with 4 ml of B-PER reagent (Pierce) containing DNaseI (0.5 µg/ml) and lysozyme (15 mg/ml). After 10-min incubation at room temperature, unbroken cells and cellular debris were removed by centrifugation at 8,000 rpm for 10 min at 4°C. Supernatant was collected and was further centrifuged at 35,000 rpm for 60 min at 4°C. The pellet was resuspended in 100 µl of 3% (w/v) sodium lauryl sarcosinate (Sigma) and incubated at room temperature for 30 min. The concentration of resulting suspensions was quantified with the BCA protein assay kit (Thermal). Two hundred micrograms of extracytoplasmic proteins were dissolved in solution (8M urea; 2M thiourea; 4% CHAPS and 80 mM DTT) and subjected to isoelectric focusing (IEF) electrophoresis with pH 4–7 carrier ampholyte for 8000 Vh. After equilibration for 15 min, the gels were transferred to the second dimension electrophoresis using 12% polyacrylamide gel and stained with the Silver Stain Plus kit (BioRad). Gel image was analyzed with ImageMasterTM 2D Platinum version 5.0 (Amersham Biosciences).

### Extraction and quantification of capsular polysaccharides (CPS)

CPS was extracted by the method described previously [56]. Five hundred microliters of bacterial culture was mixed with 100 µl of 1% Zwittergent 3–14 detergent (Sigma-Aldrich) in 100 mM citric acid (pH 2.0), and then the mixture was incubated at 50°C for 20 min. After centrifugation, 250 µl of the supernatant was transferred to a new tube, and CPS was precipitated with 1 ml of absolute ethanol. The pellet was then dissolved in 200 µl of distilled water, and a 1,200-µl volume of 12.5 mM borax (Sigma-Aldrich) in H_2_SO_4_ was added. The mixture was vigorously vortexed, boiled for 5 min, and cooled, and then 20 µl of 0.15% 3-hydroxydiphenol (Sigma-Aldrich) was added and the absorbance at 520 nm was measured. The uronic acid content was determined by the method described [57] from a standard curve of glucuronic acid (Sigma-Aldrich) and expressed as micrograms per 10^9^ CFU.

### Stress resistance assays

The *K. pneumoniae* strains to be tested were grown in LB medium at 37°C for 16 hours (8×10^8^ CFU). The stationary-phase-cultures containing 10^7^ CFU of bacteria were inoculated into LB medium or M9–0.2% glucose medium (Difco). Bacterial growth at 37°C was monitored every 1 h by measuring CFU per ml of culture with a plate counting method. For heat, H_2_O_2_, and UV treatment, approximately 5×10^8^ CFU of bacterial cells were transferred to 50°C for 10 min or to LB medium containing 200 mM H_2_O_2_ for 10 min, or exposed to UV irradiation (1 J/cm^2^), respectively. Dilutions of bacteria were spread on LB agar to determine the number of viable bacteria. Survival rates upon different stresses were expressed as the formula: (CFU after treatment/CFU before treatment) ×100%.

### Northern blotting

For Northern detection of the *rpoS or* the MicF transcript, 40 µg of total RNA isolated from *K. pneumoniae* CG43, Δ*hfq*, or Δ*hfq*-C1 was glyoxal denatured, separated on a 1% agarose gel, and then transferred onto a BrightStar Plus nylon membrane (Ambion). After UV cross-linking, the membrane was blotted with ULTRAhyb hybridization buffer (Ambion) overnight at 42°C against a *rpoS-* or MicF-specific biotin-labeled riboprobe, which was prepared using a BrightStar psoralen-biotin kit (Ambion). After a stringent wash, signals were detected with a BrightStar BioDetect kit (Ambion).

### Western blotting

Thirty micrograms of *K. pneumoniae* total proteins which were extracted from CG43S, Δ*hfq*, Δ*hfq*-C1, or Δ*hfq*-C2 with or without the induction of 0.02% arabinose were resolved by 12% of SDS-polyacrylamide gel and transferred onto a polyvinylidene difluoride (PVDF) membrane (Millipore). After blocking with 2% (w/v) skim milk at room temperature for 1 h and washes with 1×PBST, the membrane was hybridized with rabbit anti-RpoE antibody (1∶1000 dilution), rabbit anti-RpoS antibody (1∶1000 dilution), or rabbit anti-OmpA antibody (1∶1000 dilution) at 4°C for 16 hours and 5000-fold diluted HRP-conjugated anti-rabbit IgG secondary antibody was subsequently used. After stringent washes with 1×PBST, signals were detected with ECL reagent (Thermal).

## Supporting Information

Table S1 *K. pneumoniae* genes down-regulated by the absence of *hfq*.
(PDF)

Table S2 *K. pneumoniae* genes up-regulated by the absence of *hfq*.
(PDF)
