# Supplementary material for: Impact of Hfq on Global Gene Expression and Virulence in Klebsiella pneumoniae
Source: PLoS One. 2011 Jul 14;6(7):e22248. doi: 10.1371/journal.pone.0022248 (PMC3136514; doi:10.1371/journal.pone.0022248)
Supplement: Table S1 — K. pneumoniae genes down-regulated by the absence of hfq. (PDF) [file pone.0022248.s001.pdf]

Table S1. *K. pneumoniae* genes down-regulated by the absence of *hfq*.

Gene name	Product Name	Hfq-deletion		Overexpression of RpoE		Overexpression of RpoS	
		Fold change[a]		Fold change[b]		Fold change[b]	
		Average	SD	Average	SD	Average	SD
**-**	putative glycoprotein	-28.21	0.15			6.85	0.06
**lacY**	putative proton/sugar symporter	-12.98	0.21				
**rhaR**	positive regulator for rhaRS operon	-8.29	0.27				
**-**	hypothetical protein KP1_0097	-6.26	0.23				
**-**	hypothetical protein KP1_0186	-3.22	0.05				
**udp**	uridine phosphorylase	-3.09	0.04	4.25	0.02		
**fadA**	acetyl-CoA acetyltransferase	-30.37	0.21				
**fadB**	3-hydroxyacyl-CoA dehydrogenase/3-hydroxybutyryl-CoA epimerase/delta(3)-cis-delta(2)-trans-enoyl-CoA isomerase/enoyl-CoA hydratase	-11.10	0.13				
**dcuB**	anaerobic C4-dicarboxylate transporter	-7.30	0.17			7.25	0.10
**celC**	cellobiose-specific PTS family enzyme IIA component	-7.38	0.08			13.73	0.07
**-**	hypothetical protein KP1_0226	-6.24	0.03			14.10	0.03
**rsd**	regulator of sigma-D factor	-8.70	0.09				
**malG**	maltose transport system inner membrane component	-3.11	0.05				
**malF**	maltose transport system permease component	-3.65	0.12				
**-**	hypothetical protein KP1_0286	-10.36	0.08	-3.57	0.07		
**actP**	acetate permease	-23.76	0.34				
**-**	hypothetical protein KP1_0341	-64.46	0.28				
**acs**	acetyl-coenzyme A synthetase	-76.01	0.09			4.64	0.09
**-**	putative response regulator	-9.20	0.15			2.89	0.15
**-**	putative carbohydrate kinase	-10.95	0.15				
**phnN**	ATP-binding component of phosphonate transport	-5.44	0.07				
**-**	hypothetical protein KP1_0367	-5.43	0.10				
**melA**	alpha-galactosidase	-7.19	0.27				
**-**	hypothetical protein KP1_0375	-3.29	0.15	-2.86	0.06		
**aspA**	aspartate ammonia-lyase	-9.56	0.07				
**ecnB**	entericidin B	-7.27	0.06				
**blc**	outer membrane lipoprotein	-7.12	0.12				
**frdD**	fumarate reductase subunit D	-3.75	0.08			3.96	0.03
**frdC**	fumarate reductase subunit C	-5.47	0.14			3.34	0.03

**frdB**	succinate dehydrogenase	-3.53	0.05			2.94	0.08
**frdA**	fumarate reductase	-3.24	0.03			3.62	0.06
**miaA**	tRNA delta(2)-isopentenylpyrophosphate transferase	-4.03	0.04				
**hfq**	RNA-binding protein	-1008.40	0.09				
**hflX**	GTP - binding subunit of protease specific for phage lambda cII repressor	-52.22	0.38				
**hflK**	protease specific for phage lambda cII repressor	-35.82	0.05				
**hflC**	protease specific for phage lambda cII repressor	-28.19	0.13				
**aidB**	putative acyl coenzyme A dehydrogenase	-25.46	0.16			3.31	0.05
**-**	hypothetical protein KP1_0453	-17.74	0.05			15.74	0.19
**-**	hypothetical protein KP1_0454	-13.62	0.20			13.20	0.05
**ulaA**	ascorbate-specific PTS family enzyme IIC component	-3.53	0.64				
**sgaB**	putative ascorbate-specific PTS family enzyme IIB component	-3.42	0.18				
**ptxA**	putative ascorbate-specific PTS family enzyme IIA component	-2.91	0.13				
**sgaE**	L-ribulose-5-phosphate 4-epimerase	-2.83	0.08				
**cpdB**	2',3'-cyclic nucleotide 2'-phosphodiesterase/3'-nucleotidase bifunctional periplasmic precursor protein	-3.72	0.07				
**ytfQ**	putative D-ribose transport system periplasmic binding component	-9.95	0.09				
**ytfR**	putative D-ribose transport system ATP-binding component	-4.80	0.17				
**-**	putative cytoplasmic protein	-3.47	0.14	9.02	0.24		
**-**	putative cytoplasmic protein	-3.25	0.13				
**-**	putative dihydroorotase	-2.87	0.11				
**-**	putative phosphotransferase protein	-3.83	0.12				
**celF**	6-phospho-beta-glucosidase	-3.22	0.07				
**-**	putative phosphotransferase protein	-3.79	0.12				
**yjgR**	putative transport system ATP-binding component	-3.90	0.09				
**-**	hypothetical protein KP1_0551	-34.10	0.32			5.85	0.11
**-**	putative aldehyde dehydrogenase	-12.27	0.09				
**-**	hypothetical protein KP1_0553	-12.24	0.05			2.89	0.11
**iolB**	putative myo-inositol catabolism protein	-15.47	0.17			3.40	0.10
**iolD**	acetolactate synthase	-19.30	0.12				
**-**	putative NADH-dependent dehydrogenase	-13.56	0.19			3.69	0.10
**-**	putative epimerase/isomerase	-8.06	0.09			3.72	0.08
**iolE**	sugar phosphate isomerases/epimerases	-6.63	0.17			2.95	0.07

**-**	hypothetical protein KP1_0666	-2.84	0.11				
**-**	hypothetical protein KP1_0673	-3.41	0.02			3.08	0.14
**-**	hypothetical protein KP1_0674	-3.58	0.05	3.26	0.14	3.72	0.14
**-**	hypothetical protein KP1_0675	-4.36	0.05	3.10	0.17	3.78	0.11
**-**	hypothetical protein KP1_0678	-3.56	0.07			3.89	0.21
**yhhJ**	antibiotic transport system permease component	-3.96	0.15	-4.55	0.40		
**yhiH**	putative ABC-type multidrug transport system ATPase and permease component	-4.91	0.11	-3.85			
**-**	membrane fusion protein family auxiliary transport protein	-5.04	0.08				
**yjiO**	putative sugar transport protein	-4.55	0.04				
**-**	putative aldehyde dehydrogenase	-4.08	0.19				
**hpaC**	4-hydroxyphenylacetate 3-monooxygenase coupling protein	-7.76	0.09				
**hpaB**	4-hydroxyphenylacetate 3-hydroxylase	-41.60	0.19				
**hpaA**	4-hydroxyphenylacetate catabolism regulatory protein	-10.17	0.15				
**hpaX**	4-hydroxyphenylacetate permease	-18.00	0.58				
**hpaI**	2,4-dihydroxyhept-2-ene-1,7-dioic acid aldolase	-25.53	0.09				
**hpaH**	2-oxo-hepta-3-ene-1,7-dioic acid hydratase	-77.93	0.34	4.30	0.09		
**hpaF**	5-carboxymethyl-2-hydroxy-muconic acid isomerase	-57.12	0.19				
**hpaD**	3,4-dihydroxyphenylacetate 2,3-dioxygenase	-67.56	0.11			2.98	0.14
**hpaE**	5-carboxymethyl-2-hydroxymuconate semialdehyde dehydrogenase	-59.18	0.33				
**hpaG2**	4-hydroxyphenylacetate degradation bifunctional isomerase/decarboxylase C-terminal subunit	-167.67	0.14			4.43	0.27
**-**	sigma-54 dependent transcriptional regulator	-4.44	0.39				
**-**	putative PTS permease	-42.96	0.03				
**-**	putative PTS permease	-34.37	0.04				
**-**	putative PTS permease	-57.37	0.10				
**-**	putative PTS permease	-44.32	0.05				
**-**	putative glucosamine-fructose-6-phosphate aminotransferase	-72.94	0.07				
**-**	putative glucosamine-fructose-6-phosphate aminotransferase	-39.63	0.05				
**-**	putative flavoprotein monooxygenase	-14.51	0.12				
**-**	putative AraC-type regulatory protein	-15.37	0.23				
**-**	2-component transcriptional regulator	-4.48	1.00				
**mtr**	tryptophan-specific transport protein	-2.87	0.05				

**osmY**	hyperosmotically inducible periplasmic protein	-4.61	0.15	-4.76	0.17		
**ybaT**	putative amino acid/amine transport protein	-5.59	0.02				
**-**	putative Na+ dependent nucleoside transporter	-6.71	0.04			4.06	0.13
**deoC**	deoxyribose-phosphate aldolase	-4.62	0.04				
**deoA**	thymidine phosphorylase	-6.81	0.10				
**rihC**	nucleoside hydrolase	-5.53	0.12				
**-**	hypothetical protein KP1_0847	-6.82	0.09				
**citZ**	two-component response regulator for citrate	-5.54	0.12				
**citY**	two-component sensor kinase for citrate	-5.32	0.09				
**citW**	citrate/acetate antiporter	-44.47	0.14	3.72	0.06	13.73	0.04
**citX**	phosphoribosyl-dephospho-CoA transferase	-10.57	0.20				
**araC**	arabinose operon transcriptional regulator	-6.91	0.11	3.16	0.16	5.33	0.13
**yabI**	putative integral membrane protein	-2.93	0.08				
**aroP**	aromatic amino acid transport protein	-3.11	0.18				
**-**	hypothetical protein KP1_0950	-3.97	0.05				
**speE**	spermidine synthase	-2.85	0.09				
**yadI**	putative PTS enzyme II B component	-5.46	0.07				
**pulD**	pullulanase-specific type II secretion system secretin	-3.09	0.15				
**cdaR**	transcriptional regulator for gar and gud operons	-6.38	0.22				
**yaeH**	putative structural protein	-3.04	0.04				
**fadE**	acyl-CoA dehydrogenase	-25.09	0.11				
**-**	Na-translocating NADH ubiquinone oxidoreductase beta chain	-3.04	0.14				
**-**	hypothetical protein KP1_1090	-3.12	0.08				
**gabT**	4-aminobutyrate aminotransferase	-4.01	0.12				
**gabD**	NADP-dependent succinate-semialdehyde dehydrogenase I	-3.05	0.12				
**-**	hypothetical protein KP1_1103	-7.77	0.24	2.89	0.09		
**xdhC**	putative xanthine dehydrogenase Fe-S subunit	-8.78	0.15				
**-**	putative methyltransferase	-5.13	0.11				
**-**	predicted phospho-2-dehydro-3-deoxyheptonate aldolase	-4.34	0.19			3.51	0.20
**livH**	ABC transport system permease component	-4.83	0.20				
**eutC**	ethanolamine ammonia-lyase small subunit	-4.53	0.29				
**eutB**	ethanolamine ammonia-lyase large subunit	-6.83	0.22				
**-**	hypothetical protein KP1_1185	-2.88	0.03	-2.86	0.16		
**-**	hypothetical protein KP1_1187	-5.50	0.07				
**-**	hypothetical protein KP1_1193	-3.43	0.04				
**aroM**	function unknown protein of aro operon	-5.95	0.06				
**yajF**	possible NAGC-like transcriptional regulator	-8.96	0.11				

-	isochorismatase family protein	-3.07	0.19				
-	putative type II fructose-1,6-bisphosphate aldolase	-10.45	0.34				
-	putative kinase	-15.55	0.50				
-	putative sugar-specific permease	-31.64	0.15			4.58	0.09
-	putative lactose/cellobiose-specific PTS family enzyme IIB component	-25.00	0.17			5.02	0.14
-	putative phosphoenolpyruvate-dependent sugar PTS family enzyme IIA component	-31.63	0.67			8.00	0.38
**tsx**	nucleoside channel/receptor of phage T6 and colicin K	-6.49	0.13	-5.00	0.10		
**yajO**	putative NAD(P)H-dependent xylose reductase	-6.90	0.04				
-	oxidoreductase	-11.31	0.14				
-	putative IclR-family bacterial regulatory protein	-7.52	0.11				
-	hypothetical protein KP1_1266	-4.72	0.20				
**ybaY**	glycoprotein/polysaccharide metabolism	-3.13	0.10				
-	putative dTDP-glucose pyrophosphorylase	-18.28	1.06			4.11	0.06
-	putative transport ATP-binding protein	-3.22	0.08				
**yvfL**	putative maltodextrin transport permease	-4.21	0.24				
-	putative binding-protein-dependent transport systems inner membrane component	-3.47	0.29				
-	hypothetical protein KP1_1314	-3.07	0.08				
-	putative transcriptional regulator	-4.76	0.15				
-	putative GNAT-family acetyltransferase	-4.14	0.16				
-	hypothetical protein KP1_1407	-4.58	0.15				
-	hypothetical protein KP1_1421	-3.55	0.13				
**mocB**	putative rhizopine uptake ABC transport system periplasmic solute-binding protein precursor	-41.39	0.06				
-	putative ABC transporter	-17.32	0.17				
-	sugar ABC transport system permease component	-7.04	0.19				
**moaR**	putative bacterial regulatory protein	-10.21	0.23				
**moaG**	putative MFS-type transpoter	-5.57	0.29				
-	putative deacetylase	-13.95	0.77				
-	hypothetical protein KP1_1458	-3.05	1.13				
-	putative amino acid/amine transport protein	-4.20	0.33				
-	putative phosphosugar isomerases	-4.72	0.03				
-	putative inner membrane protein	-5.67	0.05				
-	putative PTS system transport protein	-3.68	0.05				
-	putative inner membrane protein	-4.26	0.10				
-	putative PTS system transport protein	-3.20	0.04				
-	putative PTS system transport protein	-5.24	0.03				

**-**	hypothetical protein KP1_1500	-3.50	0.11				
**-**	short chain dehydrogenase	-3.96	0.25				
**-**	short chain dehydrogenase/reductase family oxidoreductase	-6.88	0.11				
**-**	putative monosaccharide-transporting ATPase	-9.81	0.42				
**-**	probable ribose ABC transport system ATP-binding component	-15.33	0.23				
**-**	hypothetical protein KP1_1540	-16.51	0.10				
**-**	probable ribose ABC transport system sugar-binding component	-6.90	0.09			4.21	0.11
**-**	putative carbohydrate kinase	-18.63	0.10			3.87	0.13
**-**	putative transketolase N-terminal subunit	-7.05	0.20				
**-**	putative transketolase C-terminal subunit	-5.17	0.13	15.65	0.19		
**cstA**	carbon starvation protein	-14.70	0.21	3.02	0.16	4.46	0.05
**-**	putative short-chain alcohol dehydrogenase	-4.57	0.09				
**-**	hypothetical protein KP1_1579	-2.89	0.19				
**ybdQ**	universal stress protein G	-17.19	0.17				
**ybeL**	putative alpha helical protein	-4.15	0.08			2.84	0.04
**gltL**	glutamate/aspartate transport ATP-binding protein	-3.61	0.13				
**gltK**	glutamate/aspartate transport system permease component	-3.88	0.09			3.07	0.04
**gltJ**	glutamate/aspartate transport system permease	-3.74	0.07	3.52	0.10	4.29	0.06
**gltI**	glutamate/aspartate periplasmic binding protein	-6.71	0.04			4.37	0.19
**gltA**	citrate synthase	-6.20	0.10			3.45	0.06
**sdhC**	succinate dehydrogenase cytochrome b556 large membrane subunit	-6.52	0.04	7.16	0.04	2.93	0.04
**sdhD**	succinate dehydrogenase hydrophobic subunit	-7.55	0.06	6.44	0.03	2.91	0.04
**sdhA**	succinate dehydrogenase flavoprotein subunit	-5.56	0.13	4.03	0.06		
**sdhB**	succinate dehydrogenase catalytic subunit	-4.10	0.22				
**sucA**	2-oxoglutarate decarboxylase	-5.57	0.19	3.31	0.05		
**sucB**	dihydrolipoamide acetyltransferase	-5.90	0.20	4.81	0.04		
**sucC**	succinyl-CoA synthetase beta subunit	-4.94	0.12	4.80	0.05		
**sucD**	succinyl-CoA synthetase alpha subunit	-3.93	0.18	3.77	0.06		
**ybgS**	putative homeobox protein	-16.72	0.14				
**-**	hypothetical protein KP1_1729	-5.48	0.14	-3.33			
**-**	hypothetical protein KP1_1734	-3.97	0.10				
**-**	hypothetical protein KP1_1737	-6.54	0.16				
**-**	hypothetical protein KP1_1738	-6.88	0.18				
**uspG**	universal stress protein G	-9.09	0.17				

**-**	putative cation transport protein	-7.97	0.04				
**hutI**	imidazolonepropionase	-4.63	0.15				
**hutG**	formimionoglutamate hydrolase	-7.19	0.11				
**-**	putative urocanase	-32.43	0.06				
**hutH**	histidine ammonia lyase	-29.64	0.13			3.56	0.05
**proY**	putative amino acid permease	-12.66	0.12				
**ybhN**	putative negative regulator	-5.67	0.15				
**ybhO**	cardiolipin (CL) synthase 2	-6.80	0.11				
**ybhP**	putative DNase	-6.18	0.09				
**-**	hypothetical protein KP1_1773	-6.53	0.06	5.98	0.05		
**-**	putative LysR-family bacterial regulatory protein	-9.86	1.41				
**ybiI**	hypothetical Zinc-finger containing protein	-3.87	0.12	-4.63	0.11		
**-**	hypothetical protein KP1_1790	-3.49	0.04				
**ybiO**	putative transport protein	-3.07	0.15				
**glnH**	glutamine ABC transport system periplasmic binding component	-2.99	0.09				
**dps**	DNA protection during starvation conditions	-3.49	0.18				
**ybiW**	putative formate acetyltransferase 3	-8.21	0.08				
**ybiY**	putative pyruvate formate lyase activating enzyme	-3.57	0.11				
**cspD**	cold shock-like protein	-9.35	0.05	3.23	0.06		
**-**	putative diogenase beta subunit	-5.99	0.16				
**-**	putative aldehyde dehydrogenase	-7.52	0.37	3.34	0.27		
**-**	putative di(mono)oxygenase alpha subunit	-11.04	0.15				
**yeaV**	putative transport protein	-19.15	0.38				
**-**	putative tartrate dehydrogenase	-4.00	0.21				
**-**	probable oxidoreductase	-3.54	0.17			4.21	0.07
**-**	aldehyde dehydrogenase	-5.14	0.19				
**-**	hypothetical protein KP1_2003	-4.06	0.20				
**puuD**	probable amidotransferase subunit	-4.49	0.10				
**-**	putative glutamine synthetase	-4.91	0.19				
**ycjJ**	putative amino acid-amine transport protein	-4.69	0.14	4.09	0.12		
**agp**	glucose-1-phosphatase	-9.21	0.06				
**-**	hypothetical protein KP1_2018	-7.57	0.05				
**-**	putative transmembrane protein	-4.26	0.11				
**putA**	trifunctional transcriptional regulator/proline dehydrogenase/pyrroline-5-carboxylate dehydrogenase	-14.61	0.07				
**-**	hypothetical protein KP1_2035	-8.20	0.06	5.48	0.06		
**-**	PhoH family protein	-9.44	0.08				
**-**	hypothetical protein KP1_2053	-3.33	0.07				

**-**	triosephosphate isomerase	-3.23	0.16				
**-**	hypothetical protein KP1_2215	-12.09	0.04				
**-**	hypothetical protein KP1_2216	-10.12	0.13				
**phoC**	acid phosphatase	-6.11	0.07			8.56	0.02
**argD**	bifunctional N-succinyldiaminopimelate-aminotransferase/ acetylornithine transaminase protein	-136.14	0.64	4.08	0.10		
**astA**	arginine succinyltransferase	-81.81	0.11	4.08	0.09		
**astD**	aldehyde dehyrogenase	-105.48	0.67	3.92	0.07		
**astB**	succinylarginine dihydrolase	-65.62	0.15	4.88	0.07	2.88	0.07
**astE**	succinylglutamate desuccinylase	-77.88	0.84	6.77	0.15	2.98	0.14
**katE**	RpoS-dependent hydroperoxidase II	-5.89	0.13				
**katE**	RpoS-dependent hydroperoxidase II	-7.28	0.12				
**-**	hypothetical protein KP1_2314	-39.38	0.12			10.92	0.03
**acnA**	aconitate hydratase	-4.24	0.09				
**-**	hypothetical protein KP1_2329	-13.08	0.16				
**codB**	probable transporter	-65.18	0.18				
**-**	hypothetical protein KP1_2452	-29.59	0.14			3.87	0.04
**feaR**	2-phenylethylamine catabolism regulatory protein	-12.96	0.27				
**feaB**	phenylacetaldehyde dehydrogenase	-7.51	0.08				
**tynA**	copper-requiring tyramine oxidase	-3.10	0.14				
**paaZ**	aldehyde dehydrogenase/enoyl-CoA hydratase	-71.79	0.24				
**-**	hypothetical protein KP1_2471	-5.93	1.09				
**paaA**	phenylacetic acid degradation protein	-833.20	0.20				
**paaB**	phenylacetic acid degradation protein	-442.24	0.36				
**paaC**	phenylacetic acid degradation protein	-114.66	0.26				
**paaD**	phenylacetic acid degradation protein	-218.92	0.29				
**paaE**	probable phenylacetic acid degradation NADH oxidoreductase	-404.92	0.37	2.85	0.19		
**paaF**	probable enoyl-CoA hydratase	-204.33	0.22				
**paaG**	enoyl-CoA hydratase	-75.97	0.35	3.35	0.26		
**paaH**	3-hydroxybutyryl-CoA dehydrogenase	-53.15	0.63				
**paaI**	phenylacetic acid degradation protein	-34.42	0.50				
**paaK**	phenylacetate-CoA ligase	-3.24	0.23				
**-**	putative succinylornithine transaminase	-3.14	0.10				
**-**	arginine succinyltransferase	-3.14	0.15				
**astD**	succinylglutamic semialdehyde dehydrogenase	-3.06	0.05				
**aldA**	NAD-linked aldehyde dehydrogenase A	-2.94	0.17				
**gapA**	glyceraldehyde-3-phosphate dehydrogenase	-5.27	0.08				
**yqaE**	putative transport protein	-7.91	0.02				
**-**	hypothetical protein KP1_2515	-7.80	0.08				

**malX**	maltose/glucose-specific PTS family enzyme IIBC component	-5.65	0.32			6.09	0.15
**malI**	maltose regulon regulatory protein	-3.59	0.12				
**-**	hypothetical protein KP1_2547	-4.24	0.21				
**-**	putative fumarate lyase	-3.02	0.13				
**-**	putative 3-oxoadipate enol-lactonase II	-3.36	0.08	2.95	0.06		
**-**	hypothetical protein KP1_2570	-22.97	0.05			13.82	0.03
**-**	hypothetical protein KP1_2594	-26.90	0.09			7.22	0.03
**ynfL**	putative LysR-family transcriptional regulator	-3.52	0.11				
**ynfH**	putative dimethyl sulfoxide reductase anchor subunit	-4.33	0.06				
**dmsB**	anaerobic dimethyl sulfoxide reductase subunit B	-2.90	0.13				
**-**	hypothetical protein KP1_2617	-4.68	0.08	-3.61	0.20		
**ydeJ**	competence damage-inducible protein A	-3.92	0.11				
**ygbM**	putative epimerase/isomerase	-3.57	0.43	4.02	0.06		
**-**	ygbK domain protein	-2.95	0.36				
**-**	hypothetical protein KP1_2632	-3.94	0.56				
**lacY**	putative proton/sugar symporter	-3.58	0.58				
**lacZ**	beta-D-galactosidase	-3.72	0.30				
**-**	putative methionine synthase	-9.91	0.04	-10.63	0.11	-6.06	0.06
**-**	high-affinity branched-chain amino acid transporter periplasmic binding component	-4.01	0.39			-2.87	0.17
**-**	hypothetical protein KP1_2690	-4.01	0.08	4.18	0.06		
**-**	phosphoenolpyruvate-dependent PTS family enzyme IIA component	-6.47	0.09				
**-**	galactose-proton symport of transport system	-7.73	0.09			2.97	0.06
**-**	putative general substrate transporter	-4.07	0.32				
**-**	major facilitator family transporter	-13.71	0.50				
**-**	putative acetyl-CoA acetyltransferase	-5.72	0.08				
**fabG**	putataive short-chain dehydrogenase/reductase	-4.82	0.89				
**-**	bifunctional putative acetyl-CoA:acetoacetyl-CoA transferase: alpha subunit/beta subunit	-13.58	0.76				
**-**	putative acyl-CoA synthase	-30.80	1.03				
**-**	ribokinase	-4.79	1.03				
**-**	putative ribulose-phosphate 3-epimerase	-4.75	0.34				
**-**	putative DeoR-family bacterial regulatory protein	-3.55	0.21				
**pgtA**	phosphoglycerate transport system activator protein	-2.91	0.10				

**pgtC**	phosphoglycerate transport system regulatory protein	-6.65	0.35				
**ygaU**	putative peptidoglycan-binding protein	-7.34	0.12				
**-**	putative ABC transporter	-7.69	0.72			3.52	0.14
**-**	putative ribose/xylose/arabinose/galactoside ABC-type transport system permease component	-10.01	0.38			3.72	0.15
**-**	putative hydrolase	-4.40	0.16				
**-**	hypothetical protein KP1_2778	-6.63	0.15	4.12	0.09		
**ulaA**	ascorbate-specific PTS family enzyme IIC component	-4.37	0.18			3.02	0.07
**-**	ascorbate-specific PTS family enzyme IIA component	-2.84	0.18				
**-**	putative transporter	-9.56	0.08	4.15	0.07	2.88	0.06
**-**	putative oxidoreductase	-8.62	0.11				
**-**	oxidoreductase alpha	-2.88	0.03				
**-**	hypothetical protein KP1_2889	-4.79	0.14				
**-**	osmotically inducible protein	-6.02	0.04				
**-**	hypothetical protein KP1_2893	-3.82	0.13				
**-**	hypothetical protein KP1_2894	-3.84	0.12				
**-**	hypothetical protein KP1_2895	-3.14	0.35				
**-**	hypothetical protein KP1_2896	-3.40	0.30				
**-**	putative oxidoreductase	-3.74	0.12				
**-**	putative glycosidase	-5.10	0.19				
**-**	cellobiose/salicin/arbutin-specific PTS family enzyme IIBC component	-4.88	0.18				
**-**	putative oxidoreductase	-5.85	0.09				
**-**	hypothetical protein KP1_2926	-4.58	0.16				
**-**	NADH oxidoreductase	-3.62	0.64				
**-**	putative muconolactone delta-isomerase	-3.06	0.59				
**-**	putative transcriptional regulator	-7.22	0.23				
**narU**	nitrate extrusion protein	-12.88	0.46				
**narZ**	cryptic nitrate reductase 2 alpha subunit	-8.58	0.28				
**narY**	cryptic nitrate reductase 2 beta subunit	-3.07	0.21				
**narW**	cryptic nitrate reductase 2 delta subunit	-7.68	0.27				
**narV**	cryptic nitrate reductase 2 gamma subunit	-6.44	0.09				
**-**	putative transport protein	-15.86	0.74	5.08	0.23		
**yncG**	hypothetical GST-like protein	-3.60	0.14				
**rihA**	pyrimidine specific nucleoside hydrolase	-9.59	0.10			5.83	0.07
**-**	putative NAD-dependent aldehyde dehydrogenases	-5.10	0.21				
**ydcV**	putative transport system permease	-5.89	1.12				

	component						
**ydcU**	putative transport system permease component	-7.47	0.39				
**ydcT**	putative transport system ATP-binding component	-11.11	0.23				
**ydcS**	putative transport system periplasmic binding component	-14.74	0.27				
**ydcK**	putative LpxA-like enzyme	-5.58	0.07				
**ydcI**	putative LysR-family transcriptional regulator	-18.37	0.06	4.57	0.15		
**sodC**	superoxide dismutase	-4.11	0.03				
**ydhF**	putative NAD(P)-linked oxidoreductase	-3.26	0.10				
**cfa**	cyclopropane-fatty-acyl-phospholipid synthase	-4.43	0.06				
**-**	hypothetical protein KP1_3101	-3.00	0.03				
**-**	putative LysR-family transcriptional regulator	-2.91	0.10				
**-**	putative LysR-family transcriptional regulator	-16.57	0.35	17.21	0.08		
**-**	putative acetyl-CoA:acetoacetyl-CoA transferase alpha subunit	-10.52	0.22				
**-**	putative acetyl-CoA:acetoacetyl-CoA transferase beta subunit	-12.52	0.14				
**-**	beta-ketothiolase	-8.51	0.30	3.09	0.37		
**-**	3-hydroxybutyryl-CoA dehydrogenase	-16.60	0.44				
**-**	citrate transporter	-3.34	0.15				
**-**	short chain dehydrogenase	-3.38	0.12	3.00	0.06		
**-**	putative ABC transport system ATP-binding component	-314.99	0.17	3.87	0.13		
**-**	putative ABC transport system inner membrane permease	-1012.35	1.34	4.82	0.05	5.23	0.06
**-**	putative ABC transport system periplasmic binding component	-984.66	0.20	2.88	0.14	3.89	0.08
**-**	formate dehydrogenase H selenopolypeptide subunit	-4.36	0.10				
**mhpR**	transcriptional activator for 3-hydroxyphenylpropionate degradation	-3.76	0.05				
**sufS**	selenocysteine lyase	-4.08	0.10				
**sufD**	Fe-S cluster assembly protein	-3.14	0.10				
**sufC**	putative ATP-binding component of a transport system	-3.01	0.19				
**sufA**	iron-sulfur cluster assembly scaffold protein	-3.34	0.07				
**-**	hypothetical protein KP1_3252	-6.07	0.20	-5.94	0.35	-5.28	0.27
**-**	hypothetical protein KP1_3256	-3.09	0.13				
**ppsA**	phosphoenolpyruvate synthase	-3.27	0.18				
**yniA**	kinase-like protein	-6.82	0.08			3.21	0.04

**-**	hypothetical protein KP1_3358	-67.46	0.11			8.39	0.03
**-**	hypothetical protein KP1_3362	-8.23	0.13				
**mocB**	putative rhizopine uptake ABC transport system periplasmic solute-binding protein precursor	-51.95	0.14			2.90	0.06
**-**	putative permease of the major facilitator superfamily	-2.97	0.16				
**livM**	ABC-type high-affinity branched-chain amino acid transport system permease component	-2.88	0.49	3.25	0.13		
**livJ**	ABC-type high-affinity branched-chain amino acid transport system periplasmic binding component	-4.63	0.34				
**tdcE**	formate C-acetyltransferase	-6.10	0.16				
**tdcD**	propionate kinase	-15.52	0.09				
**tdcC**	L-threonine/L-serine permease	-19.50	0.32				
**tdcB**	threonine dehydratase	-11.68	0.43				
**tdcA**	LysR family transcriptional activator for amino acids degradation	-33.29	0.16			2.90	0.12
**treA**	periplasmic trehalase	-6.71	0.04				
**alr**	alanine racemase	-4.90	0.02				
**dadA**	D-amino acid dehydrogenase small subunit	-5.76	0.07				
**-**	hypothetical protein KP1_3434	-5.06	0.09				
**fadD**	acyl-CoA synthase	-3.14	0.13				
**-**	hypothetical protein KP1_3479	-12.99	0.26				
**-**	hypothetical protein KP1_3480	-3.32	0.05				
**pphA**	protein phosphatase 1	-4.68	0.12				
**cutC**	copper homeostasis protein	-10.80	0.10				
**yecG**	putative regulator	-3.33	0.17	-3.03	0.15		
**araF**	L-arabinose-binding periplasmic protein	-3.07	0.18			6.84	0.14
**togT**	putative oligogalacturonide transporter	-4.53	0.24	-3.48	0.54		
**amyA**	cytoplasmic alpha-amylase	-4.34	0.03				
**-**	hypothetical protein KP1_3554	-5.01	0.13				
**-**	hypothetical protein KP1_3652	-4.53	0.17				
**-**	putative non-heme chloroperoxidase	-4.59	0.15				
**sbmC**	DNA gyrase inhibitor	-6.84	0.07			2.99	0.02
**dalT**	D-arabinitol transporter	-2.88	0.15				
**-**	xylulokinase	-5.87	0.87				
**-**	D-arabinitol dehydrogenase	-3.63	0.69				
**-**	putative ribitol dehydrogenase	-38.37	0.28	2.97	0.10	7.98	0.12
**-**	ribulokinase	-38.77	0.06			5.64	0.11
**rbtT**	ribitol transporter	-10.84	0.10				
**fbaB**	class I fructose-bisphosphate aldolase	-5.13	0.11				
**yehT**	putative two-compoent regulatory system	-11.02	0.11			3.22	0.06

	response regulator						
**yehU**	putative two-compoent regulatory system sensor kinase	-8.77	0.14				
**yehY**	putative ABC-type proline/glycine betaine transport system permease component	-3.99	0.11				
**yehZ**	putative ABC-type proline/glycine betaine transport system periplasmic component	-2.88	0.08				
**-**	hypothetical protein KP1_3799	-6.06	0.18	-3.10	0.15		
**-**	putative transcriptional antiterminator	-9.45	0.10				
**mglC**	methyl-galactoside transport system permease component	-62.17	0.06	3.54	0.08		
**mglA**	methyl-galactoside transport system ATP-binding component	-31.64	0.29				
**mglB**	methyl-galactoside transport system substrate-binding component	-13.20	0.07				
**galS**	mgl operon transcriptional repressor	-60.77	0.12				
**fruA**	fructose-specific PTS family enzyme IIB'BC component	-3.92	0.10				
**yeiQ**	putative mannitol dehydrogenase	-4.37	0.09				
**spr**	putative lipoprotein	-3.15	0.06				
**ompC**	outer membrane porin protein C	-3.32	0.08	-18.38	0.09		
**glpQ**	periplasmic glycerophosphodiester phosphodiesterase	-5.96	0.06				
**glpT**	sn-glycerol-3-phosphate permease	-5.16	0.03			2.98	0.15
**glpA**	sn-glycerol-3-phosphate dehydrogenase large subunit	-5.97	0.02				
**glpB**	anaerobic glycerol-3-phosphate dehydrogenase subunit B	-4.05	0.14				
**yfaX**	putative regulator	-4.72	0.17				
**yfcG**	putative S-transferase	-5.49	0.09				
**argT**	lysine-, arginine-, ornithine-binding periplasmic protein	-3.82	0.12				
**fadJ**	3-hydroxyacyl-CoA dehydrogenase	-4.61	0.05				
**fadI**	acetyl-CoA acetyltransferase	-6.02	0.12				
**fadL**	long-chain fatty acid transport protein	-7.92	0.19	-3.20	0.36		
**-**	putative pyruvate decarboxylase	-3.92	0.08				
**mntH**	manganese transport protein	-8.43	0.09				
**nupC**	nucleoside permease	-14.39	0.17				
**yfeC**	putative negative regulator	-5.17	0.11	-2.89	0.21		
**yfeD**	putative negative regulator	-4.96	0.14				
**yfeG**	putative AraC-type regulatory protein	-3.64	0.12				
**yffI**	putative carboxysome structural protein for	-4.00	0.13	3.27	0.47		

	ethanolamine utilization						
**eutL**	ethanolamine utilization protein	-3.45	0.08				
**eutB**	ethanolamine ammonia-lyase heavy chain	-2.85	0.11				
**eutA**	putative chaperonin in ethanolamine utilization	-3.12	0.16				
**eutG**	putative transport protein in ethanolamine utilization	-6.79	0.96				
**eutJ**	ethanolamine utilization protein	-4.88	1.40				
**eutE**	ethanolamine utilization protein	-6.53	0.26				
**cchB**	detox protein	-3.41	0.09	8.31	0.17		
**cchA**	detox protein	-9.68	0.17	3.36	0.12		
**eutD**	phosphate acetyltransferase	-7.73	0.95				
**eutT**	putative cobalamin adenosyltransferase in ethanolamine utilization	-14.12	0.73				
**eutQ**	putative regulator of ethanolamine utilization	-5.08	0.15	5.69	0.20		
**eutP**	putative ethanolamine utilization protein	-5.20	0.31				
**ypfE**	putative carboxysome structural protein in ethanol utilization	-7.23	0.29				
**talA**	transaldolase	-6.31	0.13				
**tktB**	transketolase	-6.07	0.11				
**-**	putative beta-glucoside kinase	-4.09	0.07			5.45	0.30
**csiE**	stationary phase inducible protein	-47.94	0.29			5.79	0.12
**kgtP**	alpha-ketoglutarate permease	-28.13	0.08	9.06	0.06	8.45	0.05
**-**	hypothetical protein KP1_4168	-4.27	0.08				
**yfiA**	putative yhbH sigma-54 modulator	-5.68	0.10				
**-**	putative short-chain dehydrogenase/reductase	-5.56	0.08				
**aldB**	aldehyde dehydrogenase B	-7.28	0.17				
**-**	hypothetical protein KP1_4264	-6.07	0.05	-3.61	0.13		
**nrdH**	glutaredoxin-like protein	-3.34	0.30			-3.56	0.32
**hypF**	hydrogenase maturation protein	-2.98	1.05				
**hydN**	electron transport protein	-3.44	0.19	-3.63	0.20		
**hycI**	hydrogenase 3 large subunit C-terminal protease	-4.52	0.12				
**hycH**	formate hydrogenlyase maturation protein	-3.91	0.07				
**hycG**	hydrogenase 3 small subunit	-4.45	0.14				
**hycE**	hydrogenase 3 large subunit	-6.24	0.08				
**hypB**	guanine-nucleotide binding protein	-2.92	0.12	3.36	0.21		
**hypC**	hydrogenase expression/formation protein	-3.31	0.12				
**hypD**	hydrogenase expression/formation protein	-4.92	0.11				
**-**	acetolactate synthase III large subunit	-9.15	0.46				
**rpoS**	RNA polymerase sigma factor	-3.61	0.07			249.35	0.36
**garK**	glycerate kinase I	-6.00	0.14				
**gudD**	(D)-glucarate dehydratase 1	-3.27	0.09				

**fucP**	fucose permease	-6.26	1.44				
**fucI**	L-fucose isomerase	-3.65	0.65				
**fucU**	fucose operon protein	-2.84	0.43				
**fucR**	positive regulator of the fuc operon	-3.67	0.12				
**-**	hypothetical protein KP1_4440	-3.54	0.13	-2.83	0.09	-4.35	0.12
**cbiQ**	putative cobalt transport protein	-3.67	0.10				
**-**	hypothetical protein KP1_4452	-4.75	0.09				
**-**	hypothetical protein KP1_4453	-3.64	0.05				
**-**	hypothetical protein KP1_4454	-3.58	0.05				
**-**	hypothetical protein KP1_4455	-3.10	0.05				
**-**	hypothetical protein KP1_4458	-3.36	0.11				
**-**	hypothetical protein KP1_4459	-3.42	0.03				
**-**	hypothetical protein KP1_4460	-3.45	0.11				
**-**	hypothetical protein KP1_4461	-2.91	0.03				
**cbiD**	hypothetical protein KP1_4463	-3.09	0.08				
**cbiC**	hypothetical protein KP1_4464	-3.14	0.03				
**pduA**	putative propanediol utilization protein	-10.38	0.33				
**pudB**	putative propanediol utilization protein	-8.44	0.33				
**pduC**	propanediol dehydratase large subunit	-22.17	0.33				
**pduD**	propanediol dehydratase medium subunit	-3.44	0.19	5.35	0.13		
**pduE**	propanediol dehydratase small subunit	-8.83	0.20				
**pduG**	propanediol dehydratase reactivation protein	-4.83	0.34				
**pduH**	propanediol dehydratase reactivation protein	-13.42	0.25				
**pduJ**	putative propanediol utilization protein	-11.73	0.10				
**pduK**	putative propanediol utilization protein	-5.15	0.16				
**pduL**	propanediol utilization protein	-6.05	0.19				
**pduM**	propanediol utilization protein	-7.44	0.49	3.27	0.20		
**pduN**	propanediol utilization protein	-6.91	0.87	3.06	0.37		
**pduO**	propanediol utilization B12-related protein	-3.52	0.66				
**pduP**	propanediol utilization CoA-dependent propionaldehyde dehydrogenase	-4.11	0.32				
**pduQ**	propanol dehydrogenase	-4.53	0.47				
**pduS**	propanediol utilization polyhedral body protein	-7.55	0.24				
**pduT**	propanediol utilization polyhedral body protein	-6.68	0.18				
**pduU**	propanediol utilization polyhedral body protein	-11.22	0.45				
**pduV**	propanediol utilization protein	-3.97	0.50				
**-**	hypothetical protein KP1_4510	-4.27	0.13				
**lacY**	putative galactoside permease	-3.39	0.23				
**tolB**	translocation protein TolB precursor	-3.40	0.14				
**yqeF**	acetyl-CoA acetyltransferase	-9.05	0.13				
**-**	hypothetical protein KP1_4588	-13.10	0.16			3.78	0.07
**galP**	galactose-proton symport of transport system	-11.23	0.09				

**nupG**	nucleoside transport protein	-4.81	0.14			3.70	0.09
**ribB**	3,4-dihydroxy-2-butanone 4-phosphate synthase	-11.64	0.06				
**-**	hypothetical protein KP1_4770	-18.74	0.15				
**evgA**	putative LuxR-family bacterial regulatory protein	-6.05	0.13				
**evgS**	putative sensor protein EvgS1	-4.09	0.11				
**-**	hypothetical protein KP1_4776	-311.54	0.95	-5.13	0.06		
**hdeB**	hypothetical protein KP1_4777	-96.73	0.14	-4.82	0.09		
**glpF**	glycerol uptake facilitator protein	-3.30	0.60				
**dhaB2**	glycerol dehydratase	-2.88	0.19				
**dhaD**	putative glycerol dehydrogenase	-4.12	0.07	4.25	0.06		
**dhaM**	putative PTS hybrid protein	-4.02	0.12				
**ygjG**	probable ornithine aminotransferase	-3.45	0.13				
**-**	hypothetical protein KP1_4799	-92.47	0.15			3.95	0.08
**-**	hypothetical protein KP1_4800	-81.32	0.20			2.90	0.06
**yneA**	putative LacI-type transcriptional regulator	-3.37	0.14				
**ydeZ**	putative transport system permease component	-258.09	0.19			4.59	0.16
**ydeY**	putative ABC transport system permease component	-374.46	0.98			5.71	0.11
**ego**	putative ABC transport system ATP-binding component	-329.14	0.59			6.70	0.51
**ydeW**	putative SorC-family transcriptional regulator	-56.20	0.32			4.00	0.27
**ydeV**	putative kinase	-66.19	0.56			4.24	0.18
**uxaA**	altronate hydrolase	-4.04	0.13				
**uxaC**	uronate isomerase	-3.54	0.23	-4.20	0.38		
**exuT**	transport protein of hexuronates	-2.93	0.09				
**-**	hypothetical protein KP1_4824	-3.33	0.06				
**-**	hypothetical protein KP1_4825	-4.43	0.07				
**-**	hypothetical protein KP1_4826	-4.82	0.04				
**garR**	tartronate semialdehyde reductase	-4.08	0.09				
**garL**	alpha-dehydro-beta-deoxy-D-glucarate aldolase	-7.46	0.10				
**yhaU**	putative transport protein	-9.32	0.12	-3.25	0.70		
**garD**	(D)-galactarate dehydrogenase	-32.97	0.27			3.32	0.07
**yhbO**	putative intracellular proteinase	-3.46	0.15				
**-**	hypothetical protein KP1_4869	-4.92	0.20			-3.03	0.08
**-**	mannonate dehydratase	-11.89	0.05	-3.07	0.35	5.94	0.12
**-**	putative NAD(P)-binding and starvation-sensing dehydrogenase	-6.50	0.39			3.95	0.28
**nanE**	putative N-acetylmannosamine-6-phosphate 2-epimerase	-12.82	0.10			3.49	0.13

**mdh**	malate dehydrogenase	-6.38	0.10			5.58	0.04
**-**	hypothetical protein KP1_4964	-4.04	0.15				
**-**	hypothetical protein KP1_4977	-3.17	0.12				
**bfr**	bacterioferritin	-4.43	0.16	-2.95	0.08		
**-**	hypothetical protein KP1_5075	-2.93	0.11				
**pckA**	phosphoenolpyruvate carboxykinase	-15.01	0.08			7.32	0.08
**malT**	transcriptional regulator	-6.17	0.06			6.02	0.02
**glpD**	sn-glycerol-3-phosphate dehydrogenase	-12.40	0.07	-14.03	0.11	-6.68	0.09
**glgP**	glycogen phosphorylase	-9.73	0.07				
**glgA**	glycogen synthase	-8.83	0.11				
**glgC**	glucose-1-phosphate adenylyltransferase	-10.15	0.16				
**glgX**	glycogen debranching enzyme	-5.50	0.07				
**glgB**	glycogen branching enzyme	-5.23	0.11				
**ugpC**	sn-glycerol 3-phosphate transport ATP-binding component	-7.69	0.08				
**ugpE**	sn-glycerol 3-phosphate transport membrane component	-8.75	0.19				
**ugpA**	sn-glycerol 3-phosphate transporter membrane component	-7.46	0.20	2.84	0.14		
**ugpB**	sn-glycerol 3-phosphate transport periplasmic binding component	-12.07	0.16				
**-**	putative aminotransferase class-III	-3.59	0.15				
**-**	putative GntR-family bacterial regulatory protein	-3.56	0.15	3.33	0.09		
**yhhT**	putative PerM-family permease	-5.18	0.14				
**yhiO**	universal stress protein UspB	-3.38	0.12				
**uspA**	universal stress protein A	-3.66	0.08				
**yhiP**	putative POT family peptide transport protein	-6.14	0.03			3.13	0.04
**-**	hypothetical protein KP1_5213	-2.83	0.04				
**-**	hypothetical protein KP1_5215	-5.04	0.03				
**dctA**	C4-dicarboxylate transport protein	-27.65	0.09			3.58	0.05
**yiaC**	putative acetyltransferase	-4.82	0.13				
**-**	putative sugar phosphate isomerase/epimerase	-6.20	0.14			4.23	0.05
**-**	putative kinase	-7.13	0.19				
**-**	general substrate transporter	-4.04	0.12				
**tkrA**	2-hydroxyacid dehydrogenase	-3.55	0.11				
**yiaG**	putative transcriptional regulator	-4.58	0.09	-4.23	0.08		
**xylF**	D-xylose transport system substrate-binding component	-3.09	0.16				
**yibF**	putative S-transferase	-7.15	0.14				
**mtlA**	mannitol-specific PTS family enzyme II component	-3.04	0.06			3.43	0.10

**mtlD**	mannitol-1-phosphate 5-dehydrogenase	-3.42	0.08				
**-**	hypothetical protein KP1_5292	-4.88	0.14			3.13	0.03
**lldP**	L-lactate permease	-97.59	0.08	4.48	0.12	5.85	0.10
**lldR**	putative GntR-family transcriptional repressor for L-lactate utilization	-67.66	0.09	3.06	0.17	3.71	0.07
**lldD**	L-lactate dehydrogenase	-47.17	0.07			3.59	0.11
**yibK**	putative tRNA/rRNA methyltransferase	-7.18	0.09				
**glpF**	MIP family channel protein	-38.44	0.09			4.33	0.10
**glpK**	glycerol kinase	-16.97	0.08				
**gltS**	glutamate:Na+ symporter	-8.10	0.14				
**-**	putative sugar isomerase	-6.54	0.16				
**-**	hypothetical protein KP1_5388	-3.40	0.12				
**-**	putative enzyme with a phophatase-like domain	-3.09	0.09				
**ivbL**	ilvB operon leader peptide	-3.08	0.03	-3.71	0.22		
**yidF**	putative regulator	-12.60	0.07			2.98	0.04
**-**	hypothetical protein KP1_5460	-3.04	0.09				
**-**	putative alpha-glucoside-specific PTS family enzyme IIBC component	-5.33	0.15				
**-**	putative GntR-family bacterial regulatory protein	-6.48	0.06				
**-**	hypothetical protein KP1_5465	-24.65	0.07			6.11	0.03
**-**	hypothetical protein KP1_5497	-4.02	0.15				
**yieL**	putative xylanase	-3.58	0.05				

Note. *K. pneumoniae* genes whose transcript abundances determined by microarray analysis exhibited >1.5 $\log_2$ changes are shown. [a] Fold change by the deletion of *hfq* represents the transcript abundance in the Δ*hfq* strain compared with that in CG43S. [b] Fold change upon the overproduction of RpoE and RpoS represents the transcript abundance in CG43S-pYC413 and CG43S-pYC351, respectively, compared with that in CG43S-pBAD202 after 0.02% of arabinose induction at 37°C for 3 hours. Positive numbers indicate increases; negative numbers indicate decreases.
