# Supplementary material for: Impact of Hfq on Global Gene Expression and Virulence in Klebsiella pneumoniae
Source: PLoS One. 2011 Jul 14;6(7):e22248. doi: 10.1371/journal.pone.0022248 (PMC3136514; doi:10.1371/journal.pone.0022248)
Supplement: Table S2 — K. pneumoniae genes up-regulated by the absence of hfq. (PDF) [file pone.0022248.s002.pdf]

Table S2. ***K. pneumoniae*** **genes up-regulated by the absence of** ***hfq*****.**

Gene name	Product Name	Hfq-deletion		Overexpression of RpoE		Overexpression of RpoS	
		Fold change[a]		Fold change[b]		Fold change[b]	
		Average	SD	Average	SD	Average	SD
**rhaP**	bacterial inner-membrane translocator	12.15	0.09				
-	rhamnose ABC transport system ATP-binding component	3.69	0.33				
**rhaS**	rhamnose transport system substrate-binding component	3.20	0.23				
**cpxP**	periplasmic repressor of stress-related two-component regulatory system	10.10	0.07			4.29	0.19
**ppc**	phosphoenolpyruvate carboxylase	2.96	0.08	-4.86	0.09		
**yifK**	putative amino acid/amine transport protein	27.10	0.10				
**ysgA**	putative dienelactone hydrolase	3.48	0.05				
**iclR**	repressor of aceBA operon	3.39	0.19				
**pstA**	putative Na+-dependent transporter	4.90	0.45				
**pgi**	glucose-6-phosphate isomerase	3.29	0.11	-3.22	0.04		
**malM**	periplasmic protein of mal regulon	9.48	0.06				
-	prepilin peptidase dependent protein C	3.95	0.06			-4.93	0.16
-	hypothetical protein KP1_0322	9.00	0.09	3.73	0.04		
-	putative inner membrane protein	5.22	0.11				
-	putative inner membrane protein	5.96	0.13			-3.43	0.11
**gltP**	glutamate-aspartate symport protein	3.00	0.11				
-	hypothetical protein KP1_0345	4.57	0.08				
-	hypothetical protein KP1_0385	4.04	0.05				
**mgtA**	P-type Mg2+ transport ATPase	3.91	0.06			19.03	0.02
-	hypothetical protein KP1_0656	3.69	0.04				
-	hypothetical protein KP1_0694	5.01	0.16				
-	putative MarR-family bacterial regulatory protein	3.34	0.13				
-	putative secretion protein	5.66	0.12				
-	putative (R)-hydroxyglutaryl-CoA dehydratase activator	2.97	0.08				
**yjiA**	putative synthesis protein	5.59	0.07	-3.83	0.38		
-	hypothetical protein KP1_0729	8.34	0.06			3.10	0.10
**yjiY**	carbon starvation protein	7.21	0.07	-6.48	0.51	7.41	0.12
**yjjA**	putative outer membrane protein	2.95	0.12				
-	hypothetical protein KP1_0833	3.12	0.27				
**dnaK**	molecular chaperone	4.70	0.15				

**dnaJ**	chaperone protein	4.89	0.10				
**nhaA**	pH dependent Na+/H antiporter	2.87	0.09			3.03	0.05
**secA**	preprotein translocase ATPase subunit	4.40	0.12				
**ppdD**	prelipin peptidase dependent protein	3.24	0.18				
**pdhR**	transcriptional regulator of pyruvate dehydrogenase complex	4.69	0.05	-3.95	0.10		
**aceF**	dihydrolipoamide acetyltransferase	2.89	0.09	-3.88	0.06		
**gcd**	glucose dehydrogenase	3.44	0.08				
**degP**	periplasmic membrane-associated serine protease Do	6.32	0.10	34.30	0.04	3.63	0.09
**yafE**	putative biotin synthesis protein	3.20	0.16	-3.39	0.12		
**int**	CP4-like integrase	3.22	0.28	-3.00	0.20		
**sbmA**	putative ABC superfamily transporter	9.97	0.05	85.63	0.03		
**-**	hypothetical protein KP1_1190	4.21	0.06				
**tgt**	queuine tRNA-ribosyltransferase	3.44	0.07				
**glnK**	nitrogen regulatory protein P-II 2	3.87	0.20	-4.12	0.48		
**-**	hypothetical protein KP1_1296	3.70	0.13	4.41	0.09		
**htpG**	heat shock protein 90	9.00	0.05				
**ybbL**	putative ATP-binding component of a transport system	3.26	0.13	-4.65	0.08		
**ybbN**	putative thioredoxin protein	3.95	0.04				
**-**	hypothetical protein KP1_1394	4.31	0.06				
**-**	beta-lactamase domain protein	4.60	0.08				
**-**	putative regulatory protein	3.67	0.10				
**-**	putative ABC transport system periplasmic dipeptide binding component	3.01	0.08				
**dcuC**	DcuC family dicarboxylate transport protein	3.01	0.04				
**cspE**	cold shock protein	3.39	0.15	-3.13	0.07	-6.24	0.03
**-**	hypothetical protein KP1_1618	3.06	0.09				
**dacA**	D-alanyl-D-alanine carboxypeptidase	3.38	0.10				
**-**	hypothetical protein KP1_1655	17.59	0.86				
**-**	hypothetical protein KP1_1657	2.83	0.24	-3.48	0.31		
**-**	hypothetical protein KP1_1661	3.29	0.08			-3.10	0.05
**kdpC**	potassium-transporting ATPase subunit C	2.95	0.19	7.16	0.09		
**kdpB**	potassium-transporting ATPase subunit B	3.38	0.16				
**-**	hypothetical protein KP1_1671	17.23	0.06	-9.29	0.03		
**ybgH**	PTR2-family transport protein	3.06	0.14				
**-**	protein of unknown function DUF969	3.02	0.04				
**-**	protein of unknown function DUF979	2.83	0.06				
**-**	hypothetical protein KP1_1692	3.64	0.09				

**-**	putative transmembrane protein	4.36	0.18	-3.39	0.32	-3.22	0.35
**moaB**	molybdopterin biosynthesis protein B	2.99	0.11				
**moaC**	molybdenum cofactor biosynthesis protein C	2.88	0.09				
**-**	hypothetical protein KP1_1788	10.14	0.19				
**ompX**	outer membrane protein X	3.08	0.07	-8.24	0.17		
**-**	hypothetical protein KP1_1831	2.88	0.33				
**dacC**	D-alanyl-D-alanine carboxypeptidase	3.28	0.08				
**grxA**	glutaredoxin 1 redox coenzyme	3.53	0.08				
**artJ**	arginine 3rd transport system periplasmic binding component	3.86	0.10				
**hcr**	NADH oxidoreductase for HCP	4.49	0.11				
**hcp**	hydroxylamine reductase	14.60	0.09	-3.22	0.30		
**infA**	translation initiation factor IF-1	3.08	0.16			-3.28	0.04
**cydD**	cytochrome-related transport system ATP-binding component	6.68	0.17				
**trxB**	thioredoxin reductase	2.97	0.10				
**focA**	probable formate transporter	5.14	0.09				
**ompF**	outer membrane protein 1A/OmpK35 porin	23.87	0.10	-29.61	0.20		
**pncB**	nicotinate phosphoribosyltransferase	2.85	0.10				
**pyrD**	dihydroorotate dehydrogenase	3.80	0.11				
**fabA**	3-hydroxydecanoyl-ACP dehydratase	3.07	0.09				
**ycbZ**	putative ATP-dependent protease	3.18	0.13				
**yccA**	putative TEGT family carrier/transport protein	4.35	0.08	7.16	0.08	5.86	0.07
**betA**	choline dehydrogenase	4.16	0.10				
**nupC**	NUP family nucleoside transport protein	3.97	0.37				
**ndh**	respiratory NADH dehydrogenase	10.89	0.12	-6.89	0.27		
**-**	hypothetical protein KP1_2104	5.97	0.10				
**ycfS**	putative enzyme	8.07	0.22				
**ycfX**	putative NAGC-like transcriptional regulator	2.96	0.08				
**pepT**	peptidase T	3.97	0.07	-4.44	0.13		
**-**	hypothetical protein KP1_2132	2.98	0.04	-3.09	0.16	-2.85	0.15
**-**	hypothetical protein KP1_2178	5.73	0.07				
**-**	putative bacterial lipid A biosynthesis acyltransferase	5.05	0.08	16.22	0.11		
**-**	putative chitinase II	59.37	0.07				
**sppA**	protease IV, a signal peptide peptidase	2.92	0.09				
**gdhA**	glutamate dehydrogenase	3.97	0.08				
**ydjM**	LexA regulated protein	3.04	0.10				

**sohB**	putative peptidase	3.00	0.09				
**-**	hypothetical protein KP1_2451	3.25	0.04				
**ldhA**	D-lactate dehydrogenase	7.75	0.08	-3.94	0.05		
**acpD**	acyl carrier protein phosphodiesterase	5.76	0.10	-3.18	0.05		
**-**	hypothetical protein KP1_2523	4.02	0.11				
**-**	hypothetical protein KP1_2530	4.13	0.10				
**abgT**	aminobenzoyl-glutamate transport protein	10.60	0.56				
**abgB**	aminobenzoyl-glutamate utilization protein	5.27	0.28	-4.19	0.94		
**abgA**	aminobenzoyl-glutamate utilization protein	3.83	0.12				
**-**	putative tartrate:succinate antiporter	3.45	0.14				
**-**	hypothetical protein KP1_2569	3.64	0.16				
**-**	hypothetical protein KP1_2590	5.04	0.09				
**asr**	acid shock protein	8.20	0.12	-7.75	0.09	-8.83	0.10
**bioD**	dithiobiotin synthetase	9.96	0.07				
**-**	putative universal stress protein G	6.21	0.25	-3.75	0.16		
**marB**	multiple antibiotic resistance protein	5.36	0.14				
**marA**	multiple antibiotic resistance protein	4.99	0.16	3.89	0.10		
**marR**	repressor of mar operon	3.03	0.07	6.63	0.05		
		4.18	0.57			-4.29	0.59
**sotB**	sugar efflux transporter	8.30	0.04	4.50	0.04		
**yneI**	putative aldehyde dehydrogenase	6.89	0.10				
**uxaB**	tagaturonate reductase	2.88	0.14				
**-**	putative Hcp1 family type VI secretion system effector	4.86	0.28	-3.00	0.34	-3.51	0.29
**-**	putative fimbrial-like protein	3.76	0.12				
**-**	cysteine ABC transport system periplasmic substrate-binding component	4.11	0.14				
**-**	peptidoglycan synthetase	3.02	0.21				
**-**	hypothetical protein KP1_2887	7.31	0.06				
**-**	putative cation efflux system	4.68	0.09				
**smvA**	energy-dependent efflux protein for methyl viologen resistance	4.72	0.38				
**ansP**	L-asparagine permease	5.38	0.16				
**-**	hypothetical protein KP1_3000	6.18	0.72				
**tehB**	putative methyltransferase for tellurite resistance	2.93	0.12				
**-**	hypothetical protein KP1_3022	4.52	0.08				
**-**	hypothetical protein KP1_3031	3.67	0.12				

-	hypothetical protein KP1_3053	4.32	0.19				
**nemA**	N-ethylmaleimide reductase	4.84	0.10				
**ydhO**	putative lipoprotein	2.83	0.05	3.81	0.22		
**soxS**	regulatory protein	4.18	0.19	-10.70	0.19	-5.14	0.12
**ydhP**	putative transport protein	2.87	0.13				
**purR**	purine nucleotide synthesis repressor	4.00	0.14	-6.61	0.12	-3.98	0.04
-	hypothetical protein KP1_3086	7.10	0.07				
-	hypothetical protein KP1_3087	3.45	0.06				
-	hypothetical protein KP1_3088	4.34	0.09				
-	putative carbonic anhydrase	5.96	0.03				
-	putative LysR-family transcriptional regulator	14.19	0.09	-5.35	0.08		
-	acetolactate decarboxylase	18.80	0.12	-16.28	0.75	-18.92	0.14
-	acetolactate synthase large subunit	40.17	0.23	-38.02	0.72	-18.42	0.18
-	amino acid ABC transport system ATP-binding component	4.78	0.64				
-	amino acid ABC transport system periplasmic binding component	4.64	0.24	-3.06	0.20	-1.34	0.11
-	putative lavin-dependent oxidoreductase	8.42	0.08	-5.40	0.16		
-	L-lactate dehydrogenase	3.98	0.07	-4.98	0.08		
**tonB**	energy transducer	3.89	0.15				
-	hypothetical protein KP1_3296	3.89	0.10				
**oppC**	oligopeptide ABC transport system permease component	3.02	0.06				
**oppB**	oligopeptide ABC transport system permease component	4.15	0.08				
**oppA**	oligopeptide ABC transport system periplasmic binding component	7.89	0.11				
**purU**	formyltetrahydrofolate deformylase	5.08	0.10				
**narJ**	nitrate reductase 1 delta subunit	2.91	0.17				
**narK**	nitrite extrusion protein	18.56	0.29	-4.40	0.48		
**ychP**	putative invasin	4.39	0.09			-3.61	0.02
-	hypothetical protein KP1_3339	3.71	0.12				
**ychF**	putative GTP-binding protein	3.53	0.17				
-	putative nucleoprotein/polynucleotide-associated enzyme	3.17	0.06				
-	hypothetical protein KP1_3439	5.91	0.13				
**rnd**	ribonuclease D	2.93	0.09				
**pabB**	para-aminobenzoate synthase component I	4.08	0.03			-3.07	0.09
**yebN**	hypothetical protein KP1_3462	3.24	0.09				
**rrmA**	ribosomal RNA large subunit	2.95	0.04				

	methyltransferase A						
-	division-specific transpeptidase	3.72	0.12	3.36	0.17		
**yebQ**	putative transport protein	2.85	0.08	3.41	0.18		
**htpX**	heat shock protein	5.87	0.11				
-	hypothetical protein KP1_3475	3.34	0.12	-3.45	0.07		
-	hypothetical protein KP1_3490	9.00	0.07				
**yebG**	SOS regulon DNA damage-inducible protein	3.03	0.18				
**msbB**	lipid A biosynthesis lauroyl acyltransferase	3.31	0.07				
-	hypothetical protein KP1_3508	2.92	0.07				
-	hypothetical protein KP1_3536	6.81	0.14	-4.34	0.19		
**rcsA**	transcriptional activator for ctr capsule biosynthesis	2.85	0.07			-6.59	0.08
**yedR**	putative outer membrane protein N precursor	3.78	0.08				
**pagO**	putative PhoPQ-activated integral membrane protein	3.79	0.17				
-	hypothetical protein KP1_3616	11.32	0.23	-17.37	0.29	-79.35	0.23
**rmpA**	regulator of mucoid phenotype	3.81	0.16	-7.92	0.05	-20.11	0.07
-	hypothetical protein KP1_3621	3.56	0.04	-4.54	0.19	-14.24	0.06
-	hypothetical protein KP1_3656	3.02	0.11				
-	putative tellurite resistance protein	5.62	0.16				
**dacD**	serine-type D-Ala-D-Ala carboxypeptidase	8.09	0.16				
**yeeF**	putative amino acid transporter protein	3.22	0.09	3.36	0.09		
**wbbN**	putative glycosyltransferase	3.03	0.12			-3.56	0.04
**glf**	putative UDP-galactopyranose mutase	2.92	0.08			-2.86	0.15
**wbbM**	putative glycosyltransferase	4.12	0.18	-2.87	0.05	-4.75	0.03
**wcaJ**	probable CPS biosynthesis glycosyltransferase	2.84	0.05			-4.08	0.18
-	glutamate-aspartate symport protein	31.83	0.14	-4.22	0.33		
		47.17	0.30	-4.94	0.31		
-	hypothetical protein KP1_3780	4.13	0.09				
-	putative carbohydrate-selective porin	3.23	0.20	-3.04	0.32		
**yohI**	tRNA-dihydrouridine synthase	3.25	0.15	-3.01	0.25		
-	hypothetical protein KP1_3809	5.85	0.10	-3.50	0.04		
**yohK**	putative seritonin transporter	8.94	0.04				
**yeiR**	putative enzyme	4.59	0.08				
-	hypothetical protein KP1_3837	9.93	0.65	-3.69	0.45		
**rplY**	50S ribosomal protein L25	3.26	0.05			-2.98	0.15
**yfaZ**	putative porin	12.40	0.08				

**-**	hypothetical protein KP1_3923	3.22	0.07	-3.12	0.25		
**ackA**	acetate/propionate kinase	4.51	0.08				
**pta**	phosphate acetyltransferase	3.22	0.05				
		5.80	0.80	-6.38	0.09	-9.53	0.11
**-**	hypothetical protein KP1_3981	3.34	0.16			-5.87	0.13
**hemF**	coproporphyrinogen III oxidase	2.97	0.04				
**acrD**	aminoglycoside efflux pump	3.84	0.09				
**yfgF**	putative cytochrome C-type biogenesis protein	2.92	0.17				
**guaA**	bifunctional GMP synthase/glutamine amidotransferase protein	3.99	0.05	3.63	0.08		
**guaB**	inositol-5-monophosphate dehydrogenase	9.45	0.06				
**xseA**	exodeoxyribonuclease VII large subunit	4.83	0.03				
**yphH**	putative NagC-like transcriptional regulator	2.92	0.09				
**hmpA**	dihydropteridine reductase	4.30	0.07	-3.26	0.22		
**acpS**	4'-phosphopantetheinyl transferase	2.88	0.09				
**pdxJ**	pyridoxal phosphate biosynthetic protein	3.17	0.10				
**nadB**	L-aspartate oxidase	3.29	0.08				
**yfiD**	putative formate acetyltransferase	2.92	0.05	-9.13	0.15		
**-**	hypothetical protein KP1_4179	3.02	0.05				
**-**	hypothetical protein KP1_4180	6.20	0.03			-3.09	0.15
**yfiB**	iron-regulated membrane protein	4.84	0.13				
**stpA**	DNA-bending protein with chaperone activity	5.67	0.08				
**znuA**	cation ABC transport system periplasmic cation-binding component	3.76	0.24	3.12	0.34		
**-**	putative heme/hemoglobin transport protein	3.07	0.13				
**-**	hypothetical protein KP1_4392	4.07	0.40				
**-**	hemolysin F	4.44	0.20	8.57	0.05		
**ygdQ**	putative integral membrane transport protein	15.45	0.05				
**-**	hypothetical protein KP1_4551	9.85	0.11			-9.57	0.13
**-**	putative LuxR-family bacterial regulatory protein	11.27	0.05	-2.91	0.09	-14.21	0.12
**-**	hypothetical protein KP1_4554	9.16	0.23				
**mrkF**	fimbriae stability-associated protein	11.77	0.10			-3.47	0.13
**mrkD**	fimbrial adhesin protein precursor	17.96	0.08				
**mrkC**	fimbrial biogenesis outer membrane usher protein mrkC precursor	16.64	0.07			-5.25	0.09

**mrkB**	fimbrial chaperone protein mrkB precursor	26.87	0.06			-10.38	0.05
**mrkA**	type 3 fimbrial protein mrkA precursor	7.04	0.10			-2.99	0.06
**-**	hypothetical protein KP1_4563	4.77	0.17				
**scsA**	copper-sensitivity suppressor protein A	3.46	0.07				
**scsB**	copper-sensitivity suppressor protein B	4.10	0.15				
**scsC**	copper-sensitivity suppressor protein C	11.56	0.39				
**scsD**	copper-sensitivity suppressor protein D	4.57	0.15				
**serA**	D-3-phosphoglycerate dehydrogenase	3.94	0.08	-3.10	0.06		
**-**	putative aminotransferase	3.87	0.08				
**mdaB**	modulator of drug activity B	3.43	0.07				
**-**	hypothetical protein KP1_4731	4.51	0.11				
**ygiC**	putative glutathione-like synthetase	4.03	0.09				
		4.29	0.20				
**-**	hypothetical protein KP1_4745	3.14	0.05	119.43	0.06		
**-**	hypothetical protein KP1_4746	4.74	0.15	28.44	0.05		
**uppP**	undecaprenyl pyrophosphate phosphatase	4.38	0.08	34.54	0.05		
**yqjA**	putative integral membrane protein	7.76	0.04				
**-**	hypothetical protein KP1_4822	5.74	0.08				
**-**	hypothetical protein KP1_4827	5.58	0.06	-2.87	0.17		
**-**	hypothetical protein KP1_4832	2.97	0.13	-4.23	0.20		
**-**	periplasmic protein	3.33	0.21				
**yhbU**	putative collagenase	4.78	0.11				
**yhbV**	putative protease	6.09	0.11	-4.83	0.17		
**-**	hypothetical protein KP1_4893	3.13	0.15				
**-**	hypothetical protein KP1_4962	7.33	0.08	-3.21	0.12		
**-**	hypothetical protein KP1_4963	4.01	0.08				
**yhcQ**	putative membrane located multidrug resistance protein	3.97	0.06				
**ppiA**	peptidyl-prolyl cis-trans isomerase A	4.87	0.16				
**nirB**	nitrite reductase large subunit	4.37	0.11				
**nirD**	nitrite reductase small subunit	5.29	0.15	-2.86	0.31		
**ompR**	osmolarity response regulator	3.04	0.13				
**nikA**	nickel transport system periplasmic binding component	5.13	0.07				
**-**	putative phosphatase	4.34	0.15				
**yhjE**	putative transport protein	3.06	0.16				
**dppC**	dipeptide transport system permease component	3.40	0.06				
**dppB**	dipeptide transport system permease component	2.99	0.03	2.99	0.14	4.41	0.06

**yhjW**	putative transmembrane protein	17.17	0.07	13.45	0.16		
**yhjX**	putative oxalate:formate antiporter	9.68	0.22	-3.11	0.10	-3.02	0.22
**yiaD**	putative outer membrane protein	12.97	0.11	98.36	0.16		
**glyQ**	glycyl-tRNA synthetase alpha subunit	2.84	0.06				
**-**	hypothetical protein KP1_5294	3.08	0.06				
**yibO**	phosphoglyceromutase	13.64	0.09	-3.00	0.10		
**-**	hypothetical protein KP1_5307	3.56	0.06				
**waaL**	O-antigen ligase	3.15	0.07				
**nlpA**	putative lipoprotein	2.88	0.10				
**-**	hypothetical protein KP1_5396	3.60	0.14				
**-**	putative LacI-family bacterial regulatory protein	3.31	0.14	3.81	0.06		
**ibpB**	heat shock protein	8.57	0.09			3.07	0.14
**ibpA**	heat shock protein	2.93	0.04				
**yidA**	putative hydrolase of the HAD superfamily	3.67	0.12	-3.83	0.11		
**yidZ**	putative LysR-family transcriptional regulator	2.94	0.32				
**pstS**	high-affinity phosphate transport system periplasmic binding component	3.08	0.08			15.35	0.18

Note. *K. pneumoniae* genes whose transcript abundances determined by microarray analysis exhibited >1.5 log_2 changes are shown. [a] Fold change by the deletion of *hfq* represents the transcript abundance in the Δ*hfq* strain compared with that in CG43S. [b] Fold change upon the overproduction of RpoE and RpoS represents the transcript abundance in CG43S-pYC413 and CG43S-pYC351, respectively, compared with that in CG43S-pBAD202 after 0.02% of arabinose induction at 37°C for 3 hours. Positive numbers indicate increases; negative numbers indicate decreases.
